# Association between mutational subgroups, Warburg‐subtypes, and survival in patients with colorectal cancer

**DOI:** 10.1002/cam4.4968

**Published:** 2022-07-03

**Authors:** Kelly Offermans, Josien C. A. Jenniskens, Colinda C. J. M. Simons, Iryna Samarska, Gregorio E. Fazzi, Jaleesa R. M. van der Meer, Kim M. Smits, Leo J. Schouten, Matty P. Weijenberg, Heike I. Grabsch, Piet A. van den Brandt

**Affiliations:** ^1^ Department of Epidemiology, GROW School for Oncology and Reproduction Maastricht University Medical Center+ Maastricht The Netherlands; ^2^ Department of Pathology, GROW School for Oncology and Reproduction Maastricht University Medical Center+ Maastricht The Netherlands; ^3^ Pathology and Data Analytics, Leeds Institute of Medical Research at St James's University of Leeds Leeds UK; ^4^ Department of Epidemiology, Care and Public Health Research Institute (CAPHRI) Maastricht University Medical Center+ Maastricht The Netherlands

**Keywords:** colorectal cancer, oncogenes, prognosis, survival, Warburg‐effect

## Abstract

**Background:**

Previous research suggests that Warburg‐subtypes are related to potentially important survival differences in colorectal cancer (CRC) patients. In the present study, we investigated whether mutational subgroups based on somatic mutations in *RAS*, *BRAF*, *PIK3CA*, and *MET*, which are known to promote the Warburg‐effect, as well as mismatch repair (MMR) status, hold prognostic value in CRC. In addition, we investigated whether Warburg‐subtypes provide additional prognostic information, independent of known prognostic factors like TNM stage.

**Methods:**

CRC patients (*n* = 2344) from the prospective Netherlands Cohort Study (NLCS) were classified into eight mutually exclusive mutational subgroups, based on observed mutations in *RAS*, *BRAF*, *PIK3CA,* and *MET*, and MMR status: All‐wild‐type + MMR_proficient_, *KRAS*_*mut*_ + MMR_proficient_, *KRAS*_*mut*_ + *PIK3CA*_*mut*_ + MMR_proficient_, *PIK3CA*_*mut*_ + MMR_proficient_, *BRAF*_*mut*_ + MMR_proficient_, *BRAF*_*mut*_ + MMR_deficient_, other + MMR_proficient_, and other + MMR_deficient_. Kaplan–Meier curves and Cox regression models were used to investigate associations between mutational subgroups and survival, as well as associations between our previously established Warburg‐subtypes and survival within these mutational subgroups.

**Results:**

Compared to patients with all‐wild‐type + MMR_proficient_ CRC, patients with *KRAS*_mut_ + MMR_proficient_, *KRAS*_*mut*_ + *PIK3CA*_*mut*_ + MMR_proficient_, *BRAF*_*mut*_ + MMR_proficient_, or other + MMR_proficient_ CRC had a statistically significant worse survival (HR_CRC‐specific_ ranged from 1.29 to 1.88). In contrast, patients with other + MMR_deficient_ CRC had the most favorable survival (HR_CRC‐specific_ 0.48). No statistically significant survival differences were observed for the Warburg‐subtypes within mutational subgroups.

**Conclusion:**

Our results highlight the prognostic potential of mutational subgroups in CRC. Warburg‐subtypes did not provide additional prognostic information within these mutational subgroups. Future larger‐scale prospective studies are necessary to validate our findings and to examine the potential clinical utility of CRC subtyping based on mutational subgroups.

## Graphical abstract

In this large, population‐based series of CRC patients, we have shown that mutational subgroups, based on the observed mutation frequencies of *RAS* (*KRAS, NRAS, HRAS*), *BRAF, PIK3CA*, and *MET*, as well as patients’ MMR status, are associated with important differences in survival. Warburg‐subtypes did not provide additional prognostic information within these mutational subgroups.

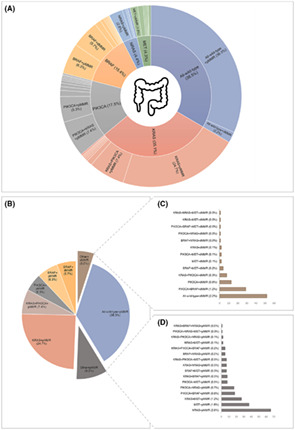

**Novelty and impact**

Our previous research suggests that Warburg‐subtypes are related to important survival differences in colorectal cancer (CRC) patients. Using data from the prospective Netherlands Cohort Study (NLCS), we investigated whether mutational subtypes based on mutations known to promote the Warburg‐effect (*RAS*, *BRAF*, *PIK3CA*, *MET*), as well as mismatch repair (MMR) status, are associated with CRC survival. Our results highlight the prognostic value of mutational subgroups, and the additional prognostic potential of Warburg‐subtypes in CRC.

## 1 INTRODUCTION

Colorectal cancer (CRC) is the third most prevalent cancer and the second leading cause of cancer‐related mortality worldwide, accounting for more than 900,000 deaths every year.^1^ Despite all efforts to identify molecular prognostic biomarkers in CRC, the tumor‐node‐metastasis (TNM) staging system remains the only clinically used prognostic factor.^2^ However, patients with the same TNM stage can have large differences in survival.^2^

Cancer cells are known to reprogram their metabolism from oxidative phosphorylation towards aerobic glycolysis, a phenomenon commonly referred to as the “Warburg‐effect”.^3^, ^4^ The Warburg‐effect is characterized by increased glucose uptake and lactate secretion in the presence of oxygen.^3^, ^4^ Since its discovery by Otto Warburg in the 1920s,^5^ the presence of the Warburg‐effect has been described in a number of different cancer types, including CRC,^6^ and has recently been proposed as one of the emerging hallmarks of cancer.^7^

Metabolic reprogramming towards the Warburg‐effect is influenced by two major oncogenic pathways: the *PI3K/AKT/mTOR* and *RAS/RAF/MEK/ERK* pathways.^8^, ^9^, ^10^, ^11^ Key genes involved in these pathways including *RAS* (*KRAS*, *NRAS*, *HRAS*), *BRAF*, *PIK3CA*, and *MET* are often mutated in human cancers,^12^, ^13^, ^14^ and these mutations have been suggested to promote the Warburg‐effect.^12^, ^13^, ^14^, ^15^ In CRC, it has previously been shown that especially *KRAS*, *BRAF*, and *PIK3CA* are frequently mutated.^10^, ^16^, ^17^ In addition, mutations in more than one of the genes (e.g., presence of *PI3KCA* mutations in combination with *RAS* or *BRAF* mutations) have been described previously.^18^, ^19^

Recently, it has become clear that *BRAF* mutations can be present in microsatellite instable (MSI) as well as in microsatellite stable (MSS) CRC.^20^ Several studies have shown that MSS *BRAF*‐mutated CRC have an aggressive phenotype (i.e., occurring at younger age, diagnosed at more advanced TNM stage, often poorly differentiated) and are associated with a poorer prognosis compared to MSI *BRAF*‐mutated CRC.^20^, ^21^ It has been described that presence of MSI ‘overrides’ the negative prognostic potential of *BRAF* mutations.^22^

Previously, we identified Warburg‐subtypes using a pathway‐based sum score after measuring the expression levels of six glycolytic proteins and transcriptional regulators indicative of the Warburg‐effect (LDHA, GLUT1, MCT4, PKM2, p53, PTEN) using immunohistochemistry (IHC).^23^ Based on this sum score, we classified CRC patients as having Warburg‐low (i.e., low probability of the presence of the Warburg‐effect), Warburg‐moderate, or Warburg‐high cancers. Our previous study suggested that Warburg‐subtypes are related to differences in survival in CRC patients, independent of known prognostic factors like TNM stage.^23^ We hypothesized that (1) mutational subgroups based on somatic mutations in *RAS*, *BRAF*, *PIK3CA*, and *MET*, which are known to promote the Warburg‐effect,^12^, ^13^, ^14^, ^15^ as well as patients' mismatch repair (MMR) status, may hold prognostic value in CRC, and (2) Warburg‐subtypes may provide additional prognostic information within these mutational subgroups, independent of known prognostic factors like TNM stage.

In this large population‐based series of CRC patients, we therefore aimed to (1) study the association between mutational subgroups based on the presence of somatic mutations in *RAS* (*KRAS*, *NRAS*, *HRAS*), *BRAF*, *PIK3CA*, and *MET*, as well as MMR status, and survival, and (2) to study the relationship between previously identified Warburg‐subtypes and survival within these mutational subgroups to examine whether Warburg‐subtypes provide additional prognostic information.

## 2 MATERIALS AND METHODS

### 2.1 Design and study population

This population‐based series of colorectal cancer (CRC) patients was derived from the prospective Netherlands Cohort Study (NLCS), which has been described in detail previously.^24^ Briefly, the NLCS was initiated in September 1986 (baseline) and included 120,852 men and women, aged 55–69 years. At baseline, all participants completed a mailed, self‐administered questionnaire on diet and other cancer risk factors.^24^ By completing and returning the questionnaire, participants agreed to participate in the study.

The NLCS was approved by the institutional review boards of the TNO Quality of Life Research Institute (Zeist, the Netherlands) and Maastricht University (Maastricht, the Netherlands). Ethical approval was obtained from the Medical Ethical Committee (METC) of Maastricht University Medical Center+.

**FIGURE 1:**
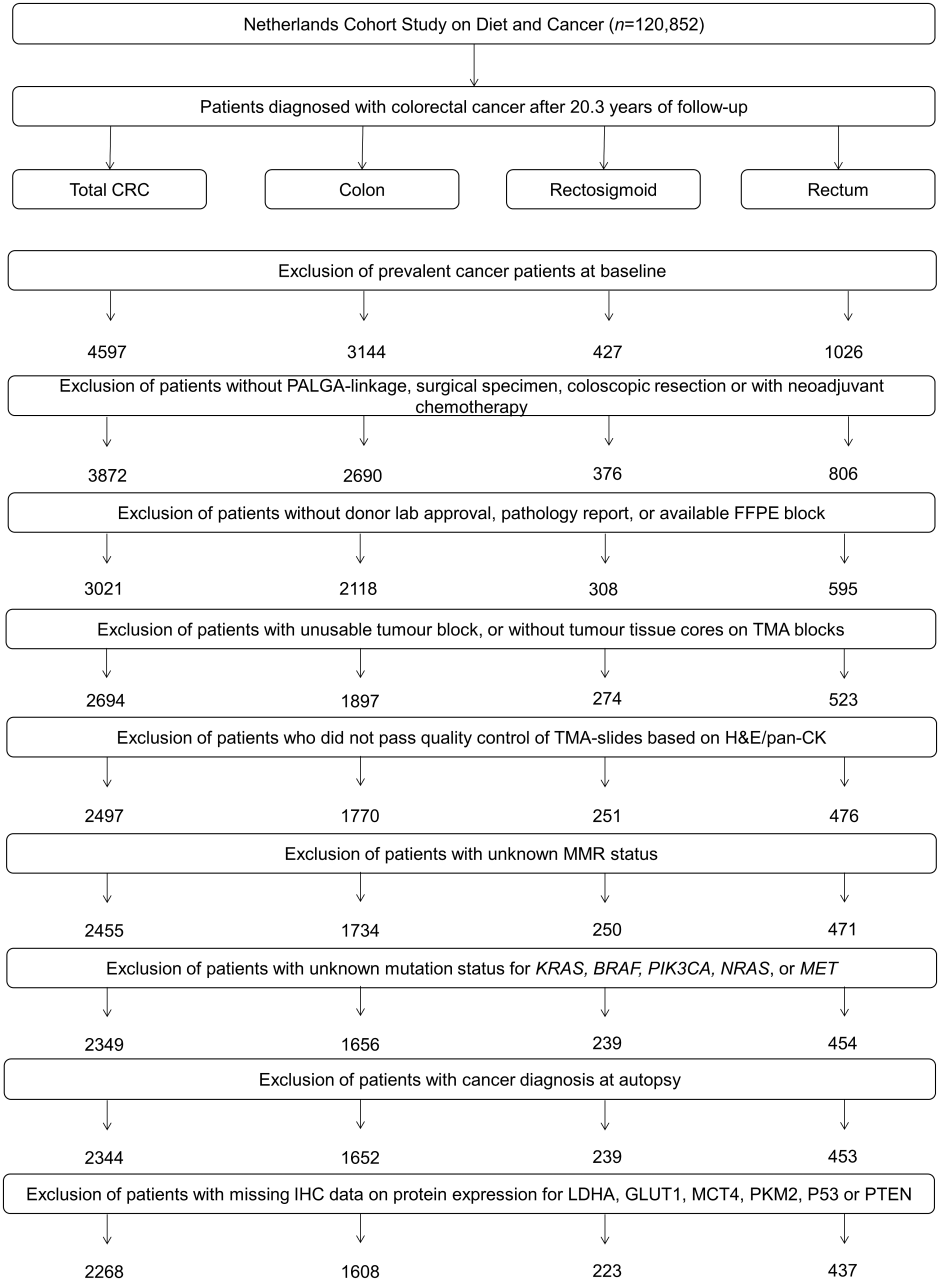
Flow diagram of the number of CRC patients available for analyses in the Netherlands Cohort Study (NLCS), 1986–2006. CRC, colorectal cancer; PALGA, Netherlands pathology database; TMA, tissue microarray.

Follow‐up for cancer incidence was established by annual record linkage with the Netherlands Cancer Registry and PALGA, the nationwide Dutch Pathology Registry,^25^, ^26^ covering 20.3 years of follow‐up (September 17, 1986 until January 1, 2007). The completeness of cancer incidence follow‐up was estimated to be >96%.^27^ After excluding patients who reported a history of cancer (excluding non‐melanoma skin cancer) at baseline, 4597 incident CRC patients were available (Figure 1).

### 2.2 Tissue collection and TMA construction

Formalin‐fixed paraffin‐embedded (FFPE) tissue blocks from CRC patients were collected as part of the Rainbow‐Tissue MicroArray (TMA) project during 2012–2017.^28^ Details of TMA construction have been described previously.^23^ In short, FFPE blocks with primary tumor and matched normal tissue of 3021 CRC patients were retrieved (78% retrieval rate) from 43 pathology laboratories throughout the Netherlands. Hematoxylin&Eosin (H&E)‐stained sections were reviewed by pathologists and areas with the highest tumor density were marked for TMA construction (TMA‐Grandmaster, 3D‐Histech, Hungary). In total, 78 TMA blocks were constructed containing three 0.6 mm cores from tumor and three from normal epithelium of 2694 CRC patients (Figure 1). In addition, two 20 μm tissue sections were cut from the tumor FFPE blocks for DNA extraction.

### 2.3 Immunohistochemistry

Five μm thick serial sections were cut from all 78 TMA blocks and subjected to either H&E staining according to a standard protocol, or subjected to immunohistochemistry (IHC) for LDHA, GLUT1, MCT4, PKM2, p53, and PTEN using an automated immunostainer (DAKO Autostainer Link 48, Glostrup, Denmark) or manual scoring protocol. Details of the primary antibodies and staining protocols have been described previously,^23^ see also Table S1. After IHC, TMA sections were scanned using the Aperio scanner (Leica Microsystems, Milton Keynes, UK) at 40x magnification at the University of Leeds (UK) Scanning Facility.

First, the presence of adenocarcinoma was confirmed for every individual core by reviewing the H&E‐stained TMA sections in combination with pan‐cytokeratin stained sections if necessary. Requiring at least one tumor core per patient, 2497 CRC patients passed quality control (Figure 1). Then, scoring of IHC was performed by three non‐pathologists (G.E. Fazzi: histology technician; K. Offermans: PhD‐student; J.C.A. Jenniskens: PhD‐student), after appropriate training.^23^, ^29^ IHC scoring protocols for all proteins, including kappa values for inter‐ and intra‐observer agreement, are shown in Table S2 and have been described in detail previously.^23^

### 2.4 DNA mismatch repair status

DNA mismatch repair (MMR) status, as a proxy for MSI status,^30^ was assessed by IHC for MLH1 and MSH2 as described previously.^23^ Briefly, cancers with loss of either MLH1 or MSH2 expression, in the presence of internal positive controls, were considered MMR deficient (dMMR). Cancers that expressed both MLH1 and MSH2 were considered MMR proficient (pMMR). Information regarding MMR status was available for 2455 CRC patients (Figure 1).

### 2.5 DNA isolation and mutational status

Two 20 μm thick FFPE tissue sections were deparaffinized manually using the Buffer ATL (Cat. No. 939011, Qiagen, Hilden, Germany), Proteinase K (Cat. No. 19131, Qiagen), and the Deparaffinization Solution (Cat. No. 19093, Qiagen), using an adapted version of the manufacturer's protocol. DNA isolation was performed using the DSP DNA Mini Kit (Cat. No. 937236, Qiagen) and the QIAsymphony® (Qiagen) instrument, following the manufacturer's protocol (Tissue_HC_200 protocol). Double‐stranded DNA (dsDNA) concentrations were quantified using the Quantus™ Fluorometer (Promega, Madison, WI, USA) with a QuantiFluor® dsDNA system (Promega).

Mutations were analyzed at the Institute for Immunology and Genetics (Kaiserslautern, Germany) using Matrix Assisted Laser Desorption Ionization Time of Flight (MALDI‐TOF) mass spectrometry and the ColoCarta Panel (Agena Bioscience, Hamburg), which screens for 32 mutations in six genes known to be commonly mutated in CRC (*KRAS*, *NRAS*, *HRAS*, *BRAF*, *PIK3CA*, *MET*; Table S3). Data analysis was performed at the Institute for Immunology and Genetics (Kaiserslautern, Germany) using MassArray Typer Analyzer software 4.0.4.20 (Sequenom) and the following cut‐offs: mutation frequency cut‐off ≥0.075; Z‐score ≥4.00; spectrum quality ≥0.750; typer peak probability ≥0.850; primer extension rate cut‐off ≥0.200.

Patients testing positive for any mutation‐specific assay were classified as mutant for the respective gene; patients with no detectable mutations were classified as wild‐type; and patients for whom testing failed or for whom equivocal results were obtained (i.e. one or more assay[s] failed and for other assays no detectable mutations were identified) were classified as having an unknown mutation status. After excluding patients with unknown mutation status for *KRAS*, *BRAF*, *PIK3CA*, *NRAS*, or *MET*, 2344 CRC patients were available for mutational subgrouping (Figure 1).

### 2.6 Mutational subgroups

**FIGURE 2:**
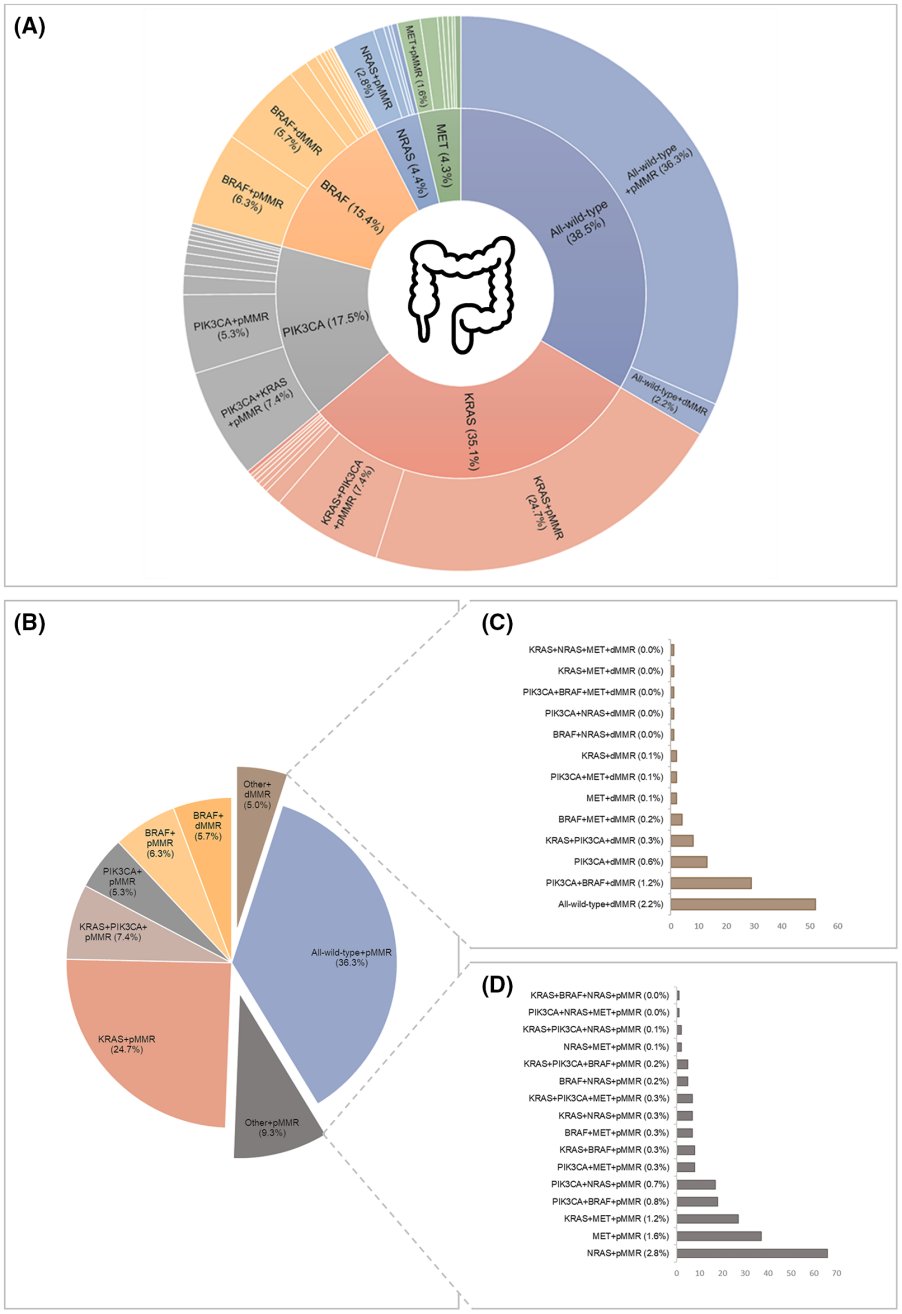
Mutation frequencies and established mutational subgroups of 2344 CRC patients within the Netherlands Cohort Study (NLCS, 1986–2006). (A) Multi‐layered pie chart showing the distribution and frequencies of genetic alterations in *KRAS*, *PIK3CA*, *BRAF*, *NRAS*, and *MET*, as well as single‐, double‐, and triple‐mutations in combination with MMR status. The inner circle shows the total mutation frequencies of *KRAS*, *PIK3CA*, *BRAF*, *NRAS*, and *MET*. The outer circle shows single‐ double‐ and triple‐ mutations which together contribute to the total mutation frequency, in combination with MMR status. Mutations with a frequency ≤1.2% are not shown. Note: Percentages do not add up to 100% because there is some degree of overlap between mutational groups (e.g., *KRAS* + *PIK3CA*). Image colon: Flaticon.com. (B) Pie chart showing the distribution and frequencies of the eight established mutational subgroups: All‐wild‐type + pMMR, *KRAS*_*mut*_ + pMMR, *KRAS*_*mut*_ + *PIK3CA*_*mut*_ + pMMR, *PIK3CA*_*mut*_ + pMMR, *BRAF*_*mut*_ + pMMR, *BRAF*_*mut*_ + dMMR, other+pMMR, and other + dMMR. (C) Histogram showing the distributions and frequencies of combinations of markers (mutational status and MMR status) that together make up the other + dMMR subgroup. (D) Histogram showing the distribution and frequencies of combinations of markers (i.e., mutational status and MMR status) that together make up the other + pMMR subgroup.

In total, 2344 CRC patients were classified into seven mutually exclusive mutational subgroups based on observed frequencies of tumor markers or combinations of tumor markers, requiring at least 100 patients per subgroup: (1) All‐wild‐type+pMMR (*n* = 851, 36.3%), (2) *KRAS*_*mut*_ + pMMR (*n* = 580, 24.7%), (3) *KRAS*_*mut*_ + *PIK3CA*_*mut*_ + pMMR (*n* = 173, 7.4%), (4) *PIK3CA*_*mut*_ + pMMR (*n* = 124, 5.3%), (5) *BRAF*_*mut*_ + pMMR (*n* = 147, 6.3%), (6) *BRAF*_*mut*_ + dMMR (*n* = 134, 5.7%), (7) other+pMMR (*n* = 218, 9.3%), and (8) other + dMMR *(n =* 117, 5.0%) (see Table S4 for details on mutational subgroups). Note, the other + pMMR group comprises all CRC patients with other (combinations of) markers and proficient MMR status (see Table S4 and Figure 2 for details). The other + dMMR subgroup includes patients with all‐wild‐type + dMMR tumors, as well as other (combinations of) markers and deficient MMR status.

### 2.7 Clinical characteristics and follow‐up

Information on patient and tumor characteristics, such as age at diagnosis, pathological (p) TNM stage, tumor location, and tumor differentiation grade was retrieved from the cancer registry or PALGA histopathology reports. Follow‐up for vital status of the CRC patients was carried out through linkage to the Central Bureau of Genealogy and the municipal population registries until December 31, 2012. Patients who were found to have CRC at autopsy were excluded (*n* = 5) (Figure 1). The cause of death was retrieved from Statistics Netherlands. CRC‐specific deaths included those with an underlying cause attributed to malignant neoplasms of the colon, rectosigmoid junction, or rectum. Vital status was available for 2343 patients, and information regarding CRC‐specific death was available for 2305 patients.

### 2.8 Warburg‐subtypes

The process of combining multiple core‐level scores of proteins involved in the Warburg‐effect (LDHA, GLUT1, MCT4, PKM2, p53, or PTEN) into patient‐level Warburg‐subtypes has been described previously.^23^ Briefly: (1) Scores from individual observers were combined into a “combination score” if the same score was given by at least two observers; (2) remaining discrepancies were either resolved by consensus agreement or an experienced pathologist determined the final score; (3) the final scores of all available tumor cores were averaged and the value was rounded to the nearest scoring category to obtain a patient‐level score; (4) the average scores per patient were categorized as low, moderate, or high protein expression; (5) the expression levels of all six proteins were combined into a pathway‐based sum score (range 0–12); (6) based on the sum score, 2268 CRC patients were categorized into the “Warburg‐low” (sum score 0–3, *n* = 646, 28.5%), “Warburg‐moderate” (sum score 4–5, *n* = 820, 36.2%) or “Warburg‐high” subtype (sum score 6–12, *n* = 802, 35.4%) (Figure 1).

### 2.9 Statistical analyses

Descriptive statistics and frequency distributions were calculated for clinical characteristics. Differences between mutational subgroups were evaluated using Chi‐square for categorical variables and Kruskal–Wallis tests for continuous variables. The primary endpoints of the current study were CRC‐specific survival, defined as the time from CRC diagnosis to CRC‐related death or end of follow‐up, and overall survival, defined as the time from CRC diagnosis to death from any cause or end of follow‐up. Because of the limited number of events in the later period with follow‐up of more than 10 years (CRC‐specific deaths: *n* = 33, 3.3%; overall deaths: *n* = 266, 14.9%), survival analyses were restricted to 10 years of follow‐up. The relationship between mutational subgroups and CRC‐specific or overall survival was estimated using Kaplan–Meier curves and Wilcoxon tests. Hazard ratios (HRs) and 95% confidence intervals (CIs) were estimated using Cox proportional hazards regression. In addition, analyses were performed stratifying CRC patients by pTNM stage or tumor location. Furthermore, the relationship between Warburg‐subtypes and CRC‐specific or overall survival within mutational subgroups was examined.

The proportional hazards assumption was tested using the scaled Schoenfeld residuals,^31^ by evaluating‐log transformed survival curves or by introducing time‐covariate interactions into the models. HRs were adjusted for a set of a priori selected prognostic factors: age at diagnosis, sex, tumor location, pTNM stage, differentiation grade, and adjuvant therapy. A separate category (‘unknown’) was used for patients with unknown clinical information regarding pTNM stage, differentiation grade, or adjuvant therapy to enable inclusion of these patients in the Cox proportional hazards models.

Disease stage was based on the pTNM classification according to the edition valid at the time of cancer diagnosis (Table S5) resulting in the use of five different TNM editions (UICC TNM edition 3–6). However, the main TNM stage groupings (I/II/III/IV) have remained essentially unchanged.^32^ Year of diagnosis and pTNM version were considered as potential confounders. Both variables were not included in the final models because they did not introduce a ≥ 10% change in HRs.

In sensitivity analyses, we repeated analyses after excluding CRC patients with unknown clinical information regarding pTNM stage, differentiation grade or adjuvant therapy (*n* = 247).

All analyses were conducted in Stata Statistical Software: Release 16 (StataCorp., College Station, TX). *p*‐values <0.05 were considered significant.

## 3 RESULTS

After quality control and excluding patients with missing information on *KRAS*, *PIK3CA*, *BRAF*, *NRAS*, or *MET* mutational status (*n* = 117) or MMR status (*n* = 279), 2344 CRC patients were available for analyses in the current study.

### 3.1 Mutation frequencies

All‐wild‐type cancers were identified in 903 (38.5%) CRC patients (Figure 2A). The majority of CRC patients (*n* = 1441, 61.5%) had at least one mutation in one of the investigated genes. *KRAS*, *BRAF*, *PIK3CA*, *NRAS* or *MET* were mutated in 35.1%, 15.4%, 17.5%, 4.4%, and 4.3% of CRC, respectively (Figure 2A). Mutations in *HRAS* were not observed. *KRAS*, *BRAF*, *PIK3CA*, *NRAS*, and *MET* were exclusively mutated in 24.8%, 12.0%, 5.8%, 2.8%, and 1.7% of CRC, respectively (Figure 2A). Two or more genes were mutated in 336 (14.3%) CRC patients. Co‐existing mutations in *KRAS* and *BRAF* were rare (*n* = 14, 0.6%). The most frequently observed double mutation included *KRAS* and *PIK3CA* (*n* = 181, 7.7%), whereas other double mutations were observed in less than 2% of CRC. Triple mutations were rare (*n* = 18, 0.8%). MMR deficiency (dMMR) was observed in 251 (10.7%) CRC patients. The majority of patients with dMMR CRC had a *BRAF* mutation (*n* = 134, 53.4%) or were all‐wild‐type (*n* = 52, 20.7%).

### 3.2 Mutational subgroups

Based on the observed single‐, double‐, or triple‐mutation frequencies and MMR status, CRC patients were classified into eight mutually exclusive mutational subgroups, requiring at least 100 patients per subgroup, as: (1) All‐wild‐type + pMMR (*n* = 851, 36.3%), (2) *KRAS*_*mut*_ + pMMR (*n* = 580, 24.7%), (3) *KRAS*_*mut*_ + *PIK3CA*_*mut*_ + pMMR (*n* = 173, 7.4%), (4) *PIK3CA*_*mut*_ + pMMR (*n* = 124, 5.3%), (5) *BRAF*_*mut*_ + pMMR (*n* = 147, 6.3%), (6) *BRAF*_*mut*_ + dMMR (*n* = 134, 5.7%), (7) other + pMMR (*n* = 218, 9.3%), and (8) other + dMMR *(n =* 117, 5.0%) (Figure 2B). The other + dMMR subgroup largely consisted of patients with all‐wild‐type CRC or patients with mutations in *BRAF* and/or *PIK3CA* (Figure 2C), whereas the other + pMMR subgroup mainly consisted of patients with mutations in *RAS (NRAS*, *KRAS)* and/or *MET* (Figure 2D).

Clinical characteristics of each mutational subgroup are shown in Table 1. Mutational subgroups differed significantly with respect to age at diagnosis, sex, tumor location, pTNM stage, tumor extension (pT), lymph node involvement (pN), differentiation grade, and adjuvant therapy.

**TABLE 1:** . Clinical characteristics of colorectal cancer patients within the Netherlands Cohort Study (NLCS, 1986–2006) according to mutational subgroup (*n* = 2344)

	Total	Mutational subgroups								
		All‐wild‐type +pMMR	*KRAS*_*mut*_ +pMMR	*KRAS*_*mut*_ +*PIK3CA*_*mut*_ +pMMR	*PIK3CA*_*mut*_ +pMMR	*BRAF*_*mut*_		Other		*p‐value* *
						*BRAF*_*mut*_ +pMMR	*BRAF*_*mut*_ +dMMR	Other +pMMR	Other +dMMR	
Number of patients, *n* (%)	2344	851 (36.3)	580 (24.7)	173 (7.4)	124 (5.3)	147 (6.3)	134 (5.7)	218 (9.3)	117 (5.0)	
Age at diagnosis in years, median (range)	74.0 (55.0–89.0)	74.0 (55.0–89.0)	74.0 (56.0–89.0)	74.0 (60.0–88.0)	72.0 (58.0–84.0)	75.0 (56.0–88.0)	76.0 (62.0–86.0)	73.0 (56.0–87.0)	74.0 (57.0–87.0)	<0.001^†^
Sex, *n* (%)										
Men	1311 (55.9)	542 (63.7)	311 (53.6)	94 (54.3)	79 (63.7)	62 (42.2)	41 (30.6)	129 (59.2)	53 (45.3)	<0.001
Women	1033 (44.1)	309 (36.3)	269 (46.4)	79 (45.7)	45 (36.3)	85 (57.8)	93 (69.4)	89 (40.8)	64 (54.7)	
Tumor location, *n* (%)										
Colon	1652 (70.5)	515 (60.5)	387 (66.7)	133 (76.9)	91 (73.4)	132 (89.8)	132 (98.5)	149 (68.4)	113 (96.6)	<0.001
Rectosigmoid	239 (10.2)	116 (13.6)	74 (12.8)	13 (7.5)	11 (8.9)	3 (2.0)	1 (0.8)	20 (9.2)	1 (0.9)	
Rectum	453 (19.3)	220 (25.9)	119 (20.5)	27 (15.6)	22 (17.7)	12 (8.2)	1 (0.8)	49 (22.5)	3 (2.6)	
pTNM stage, *n* (%)										
I	459 (19.6)	194 (22.8)	119 (20.5)	29 (16.8)	18 (14.5)	10 (6.8)	19 (14.2)	52 (23.9)	18 (15.4)	<0.001
II	877 (37.4)	305 (35.8)	185 (31.9)	74 (42.8)	53 (42.7)	47 (32.0)	65 (48.5)	75 (34.4)	73 (62.4)	
III	614 (26.2)	220 (25.9)	157 (27.1)	40 (23.1)	30 (24.2)	59 (40.1)	37 (27.6)	51 (23.4)	20 (17.1)	
IV	330 (14.1)	102 (12.0)	105 (18.1)	27 (15.6)	18 (14.5)	27 (18.4)	10 (7.5)	35 (16.1)	6 (5.1)	
Unknown	64 (2.7)	30 (3.5)	14 (2.4)	3 (1.7)	5 (4.0)	4 (2.7)	3 (2.2)	5 (2.3)	–	
Tumor extension (pT), *n* (%)										
T1	102 (4.4)	48 (5.6)	27 (4.7)	10 (5.8)	4 (3.2)	1 (0.7)	2 (1.5)	10 (4.6)	–	<0.001
T2	439 (18.7)	181 (21.3)	116 (20.0)	23 (13.3)	16 (12.9)	13 (8.8)	17 (12.7)	53 (24.3)	20 (17.1)	
T3	1511 (64.5)	522 (61.3)	373 (64.3)	117 (67.6)	89 (71.8)	101 (68.7)	94 (70.2)	129 (59.2)	86 (73.5)	
T4	221 (9.4)	67 (7.9)	46 (7.9)	20 (11.6)	10 (8.1)	27 (18.4)	19 (14.2)	21 (9.6)	11 (9.4)	
Unknown	71 (3.0)	33 (3.9)	18 (3.1)	3 (1.7)	5 (4.0)	5 (3.4)	2 (1.5)	5 (2.3)	‐	
Lymph node involvement (pN), *n* (%)										
N0	1212 (51.7)	444 (52.2)	280 (48.3)	95 (54.9)	63 (50.8)	54 (36.7)	77 (57.5)	119 (54.6)	80 (68.4)	<0.001
N+	853 (36.4)	297 (34.9)	234 (40.3)	59 (34.1)	40 (32.3)	81 (55.1)	44 (32.8)	75 (34.4)	23 (19.7)	
Unknown	279 (11.9)	110 (12.9)	66 (11.4)	19 (11.0)	21 (16.9)	12 (8.2)	13 (9.7)	24 (11.0)	14 (12.0)	
Differentiation grade, *n* (%)										
Well	198 (8.5)	79 (9.3)	57 (9.8)	15 (8.7)	11 (8.9)	10 (6.8)	4 (3.0)	17 (7.8)	5 (4.3)	<0.001
Moderate	1528 (65.2)	597 (70.2)	381 (65.7)	122 (70.5)	86 (69.4)	69 (46.9)	66 (49.3)	150 (68.8)	57 (48.7)	
Poor/undifferentiated	412 (17.6)	101 (11.9)	81 (14.0)	22 (12.7)	18 (14.5)	59 (40.1)	53 (39.6)	33 (15.1)	45 (38.5)	
Unknown	206 (8.8)	74 (8.7)	61 (10.5)	14 (8.1)	9 (7.3)	9 (6.1)	11 (8.2)	18 (8.3)	10 (8.6)	
Adjuvant therapy, *n* (%)										
No	1830 (78.1)	654 (76.9)	434 (74.8)	142 (82.1)	94 (75.8)	121 (82.3)	121 (90.3)	158 (72.5)	106 (90.6)	<0.001
Yes	494 (21.1)	186 (21.9)	139 (24.0)	31 (17.9)	29 (23.4)	26 (17.7)	13 (9.7)	59 (27.1)	11 (9.4)	
Unknown	20 (0.9)	11 (1.3)	7 (1.2)	–	1 (0.8)	–	–	1 (0.5)	–	

Abbreviations: pMMR, mismatch repair proficient; dMMR, mismatch repair deficient; TNM, tumor‐node‐metastasis; N0, no lymph node metastasis; N+ one or more lymph node metastases.

^*^ *p*‐value for the χ^2^ test, unless otherwise specified.

^†^ *p*‐value for the Kruskall–Wallis test.

Patients with *BRAF*_mut_ CRC had the highest median age at diagnosis (*p* < 0.001) and were more often women (*p* < 0.001), particularly those with *BRAF*_*mut*_ + dMMR CRC. *BRAF*_mut_ cancers were almost exclusively located in the colon (*BRAF*_*mut*_ + pMMR: 89.8%, *BRAF*_*mut*_ + dMMR: 98.5%, *p* < 0.001). In contrast, all‐wild‐type + pMMR cancers were more frequently located in the rectum compared to other mutational subgroups (*p* < 0.001).

Patients with *BRAF*_*mut*_ + pMMR, *KRAS*_*mut*_ + pMMR, *KRAS*_*mut*_ + *PIK3CA*_*mut*_ + pMMR, or other + dMMR CRC were more likely to be diagnosed with advanced pTNM stage (*p* < 0.001). Patients with *BRAF*_*mut*_ + pMMR CRC more frequently had a higher depth of invasion (pT, *p* < 0.001) and lymph node involvement (pN+, *p* < 0.001). Patients with *BRAF*_mut_ or other + dMMR CRC were more often diagnosed with poorly differentiated cancers (*p* < 0.001). Lastly, patients with *BRAF*_*mut*_ + dMMR and other + dMMR CRC least often received adjuvant therapy (*p* < 0.001).

### 3.3 Survival of CRC patients within mutational subgroups

The median (range) follow‐up time since diagnosis was 4.86 years (0.0027–25.99 years). Survival analyses were restricted to 10 years of follow‐up. During these first 10 years of follow‐up, 1522 (64.9%) deaths were observed, of which 961 (63.1%) were CRC‐related deaths.

**FIGURE 3:**
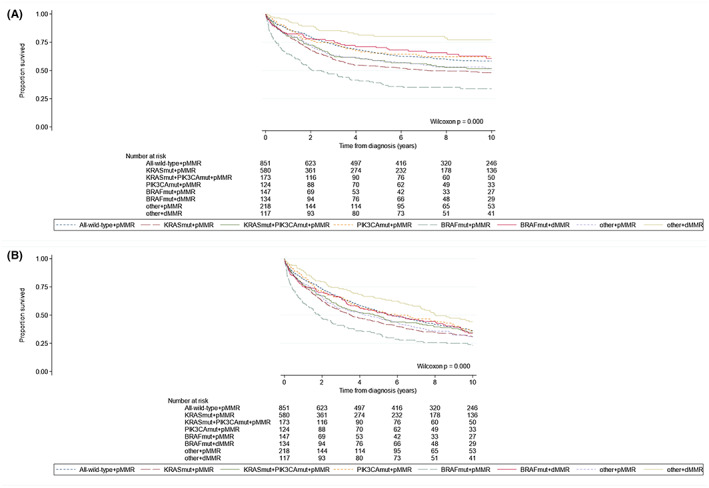
Kaplan–Meier curves according to mutational subgroups (i.e., all‐wild‐type + pMMR, *KRAS*_*mut*_ + pMMR, *KRAS*_*mut*_ + *PIK3CA*_*mut*_ + pMMR, *PIK3CA*_*mut*_ + pMMR, *BRAF*_*mut*_ + pMMR, *BRAF*_*mut*_ + dMMR, other+pMMR, and other+dMMR) in colorectal cancer patients within the Netherlands Cohort Study (NLCS, 1986–2006), showing (A) CRC‐specific survival (median survival times: *KRAS*_*mut*_ + pMMR, 7.16 years and *BRAF*_*mut*_ + pMMR, 2.48 years) and (B) overall survival (median survival times: All‐wild‐type + pMMR, 5.73 years; *KRAS*_*mut*_ + pMMR, 3.49 years; *KRAS*_*mut*_ + *PIK3CA*_*mut*_ + pMMR, 4.79 years; *PIK3CA*_*mut*_ + pMMR, 5.91 years; *BRAF*_*mut*_ + pMMR, 1.83 years; *BRAF*_*mut*_ + dMMR, 5.46 years; other + pMMR, 4.25 years; other + dMMR, 8.04 years).

Univariable Kaplan–Meier curves showed statistically significant survival differences between patients for the different mutational subgroups (Figure 3). The poorest CRC‐specific and overall‐survival was observed for patients with *BRAF*_*mut*_ + pMMR CRC, followed by *KRAS*_*mut*_ + pMMR CRC, *KRAS*_*mut*_ + *PIK3CA*_*mut*_ + pMMR, or other + pMMR CRC (Figure 3). Multivariable‐adjusted Cox‐regression models showed that patients with *KRAS*_*mut*_ + pMMR CRC, *KRAS*_*mut*_ + *PIK3CA*_*mut*_ + pMMR, *BRAF*_*mut*_ + pMMR, or other + pMMR CRC had a statistically significant worse CRC‐specific and/or overall survival compared to patients with all‐wild‐type + pMMR CRC (Table 2). Patients with *BRAF*_*mut*_ + pMMR CRC had the poorest survival (HR_CRC‐specific_ 1.88; 95% CI 1.48–2.40 and HR_overall_ 1.46; 95% CI 1.18–1.81), followed by patients with *KRAS*_*mut*_ + pMMR CRC (HR_CRC‐specific_ 1.34; 95% CI 1.14–1.58 and HR_overall_ 1.19; 95% CI 1.05–1.36), other + pMMR (HR_CRC‐specific_ 1.32; 95% CI 1.05–1.67 and HR_overall_ 1.26; 95% CI 1.05–1.52), and *KRAS*_*mut*_ + *PIK3CA*_*mut*_ + pMMR CRC (HR_CRC‐specific_ 1.29; 95% CI 1.00–1.66 and HR_overall_ 1.11; 95% CI 0.91–1.37) (Table 2). Patients with other + dMMR CRC showed the most favorable CRC‐specific and overall survival (HR_CRC‐specific_ 0.48; 95% CI 0.31–0.74 and HR_overall_ 0.73; 95% CI 0.56–0.96).

**TABLE 2:** Univariable and multivariable‐adjusted hazard ratios for associations between mutational subgroups and survival of colorectal cancer patients within the Netherlands Cohort Study (NLCS, 1986–2006), stratified on tumor location (i.e., colon, rectosigmoid, rectum)

	*N*	CRC‐specific survival			Overall survival		
		CRC deaths *n* (%)	HR (95% CI)		Deaths *n* (%)	HR (95% CI)	
			Univariable	Multivariable‐adjusted*		Univariable	Multivariable‐adjusted*
Colorectal							
All‐wild‐type + pMMR	851	316 (37.1)	1.00 (ref)	1.00 (ref)	532 (62.5)	1.00 (ref)	1.00 (ref)
*KRAS*_*mut*_ + pMMR	580	274 (47.2)	1.42 (1.21–1.67)	1.34 (1.14–1.58)	395 (68.1)	1.24 (1.09–1.41)	1.19 (1.05–1.36)
*KRAS*_*mut*_ + *PIK3CA*_*mut*_ + pMMR	173	76 (43.9)	1.27 (0.98–1.63)	1.29 (1.00–1.66)	111 (64.2)	1.10 (0.90–1.35)	1.11 (0.91–1.37)
*PIK3CA*_*mut*_ + pMMR	124	43 (34.7)	0.93 (0.68–1.28)	0.92 (0.67–1.26)	78 (62.9)	1.00 (0.79–1.27)	1.02 (0.80–1.30)
*BRAF*_*mut*_ + pMMR	147	91 (61.9)	2.28 (1.81–2.89)	1.88 (1.48–2.40)	112 (76.2)	1.72 (1.40–2.11)	1.46 (1.18–1.81)
*BRAF*_*mut*_ + dMMR	134	44 (32.8)	0.92 (0.67–1.26)	0.92 (0.66–1.29)	84 (62.7)	1.05 (0.83–1.32)	0.98 (0.77–1.25)
Other + pMMR	218	94 (43.1)	1.26 (1.00–1.59)	1.32 (1.05–1.67)	147 (67.4)	1.19 (0.99–1.43)	1.26 (1.05–1.52)
Other + dMMR	117	23 (19.7)	0.48 (0.32–0.74)	0.48 (0.31–0.74)	63 (53.8)	0.77 (0.59–1.00)	0.73 (0.56–0.96)
Colon							
All‐wild‐type + pMMR	515	202 (39.2)	1.00 (ref)	1.00 (ref)	337 (65.4)	1.00 (ref)	1.00 (ref)
*KRAS*_*mut*_ + pMMR	387	183 (47.3)	1.34 (1.09–1.63)	1.30 (1.06–1.60)	264 (68.2)	1.16 (0.99–1.37)	1.16 (0.99–1.37)
*KRAS*_*mut*_ + *PIK3CA*_*mut*_ + pMMR	133	58 (43.6)	1.20 (0.89–1.60)	1.24 (0.92–1.66)	85 (63.9)	1.05 (0.83–1.33)	1.10 (0.87–1.40)
*PIK3CA*_*mut*_ + pMMR	91	31 (34.1)	0.85 (0.58–1.24)	0.82 (0.56–1.21)	56 (61.5)	0.91 (0.69–1.21)	0.92 (0.69–1.23)
*BRAF*_*mut*_ + pMMR	132	80 (60.6)	2.07 (1.60–2.68)	1.83 (1.40–2.40)	100 (75.8)	1.59 (1.27–1.99)	1.46 (1.16–1.84)
*BRAF*_*mut*_ + dMMR	132	44 (33.3)	0.86 (0.62–1.20)	0.94 (0.67–1.33)	83 (62.9)	0.98 (0.77–1.24)	0.99 (0.77–1.28)
Other + pMMR	149	67 (45.0)	1.25 (0.95–1.65)	1.33 (1.01–1.76)	105 (70.5)	1.20 (0.97–1.50)	1.28 (1.03–1.60)
Other + dMMR	113	21 (18.6)	0.42 (0.27–0.66)	0.45 (0.29–0.72)	60 (53.1)	0.70 (0.53–0.93)	0.72 (0.54–0.96)
Rectosigmoid							
All‐wild‐type + pMMR	116	36 (31.0)	1.00 (ref)	1.00 (ref)	73 (62.9)	1.00 (ref)	1.00 (ref)
*KRAS*_*mut*_ + pMMR	74	33 (44.6)	1.54 (0.96–2.47)	1.38 (0.84–2.28)	55 (74.3)	1.33 (0.93–1.89)	1.23 (0.85–1.77)
*KRAS*_*mut*_ + *PIK3CA*_*mut*_ + pMMR	13	5 (38.5)	1.16 (0.45–2.95)	1.55 (0.60–4.03)	7 (53.8)	0.77 (0.36–1.68)	0.87 (0.40–1.91)
*PIK3CA*_*mut*_ + pMMR	11	1 (9.1)	0.24 (0.03–1.77)	0.35 (0.05–2.63)	5 (45.5)	0.57 (0.23–1.41)	0.87 (0.35–2.19)
*BRAF*_*mut*_ + pMMR	3	2 (66.7)	2.42 (0.58–10.07)	2.45 (0.55–10.81)	2 (66.7)	1.25 (0.31–5.08)	1.01 (0.24–4.27)
*BRAF*_*mut*_ + dMMR	1	0 (0.0)	–	–	1 (100.0)	–	–
Other + pMMR	20	9 (45.0)	1.47 (0.71–3.06)	1.71 (0.76–3.84)	13 (65.0)	1.03 (0.57–1.85)	1.33 (0.70–2.53)
Other + dMMR	1	0 (0.0)	–	–	1 (100.0)	–	–
Rectum							
All‐wild‐type + pMMR	220	78 (35.5)	1.00 (ref)	1.00 (ref)	122 (55.5)	1.00 (ref)	1.00 (ref)
*KRAS* + pMMR	119	58 (48.7)	1.61 (1.14–2.26)	1.51 (1.06–2.14)	76 (63.9)	1.36 (1.02–1.81)	1.29 (0.96–1.73)
*KRAS* + *PIK3CA* + pMMR	27	13 (48.1)	1.40 (0.78–2.52)	1.32 (0.73–2.39)	19 (70.4)	1.32 (0.82–2.14)	1.21 (0.74–1.97)
*PIK3CA* + pMMR	22	11 (50.0)	1.64 (0.87–3.09)	1.61 (0.85–3.07)	17 (77.3)	1.69 (1.01–2.80)	1.62 (0.97–2.73)
*BRAF* + pMMR	12	9 (75.0)	2.94 (1.47–5.86)	2.32 (1.14–4.69)	10 (83.3)	2.16 (1.13–4.12)	1.70 (0.88–3.27)
*BRAF* + dMMR	1	0 (0.0)	–	–	0 (0.0)	–	–
Other + pMMR	49	18 (36.7)	1.12 (0.67–1.87)	1.13 (0.67–1.90)	29 (59.2)	1.16 (0.77–1.73)	1.12 (0.74–1.70)
Other + dMMR	3	2 (66.7)	1.97 (0.48–8.03)	4.12 (0.99–17.21)	2 (66.7)	1.31 (0.32–5.31)	2.46 (0.60–10.09)

Abbreviations: CRC, colorectal cancer; CI, confidence interval; HR, hazard ratio; pMMR, mismatch repair proficient; dMMR, mismatch repair deficient.

* Adjusted for age at diagnosis (years), sex (men/women), tumor location (colon/rectosigmoid/rectum), pTNM stage (I/II/III/IV/unknown), differentiation grade (well/moderate/poor/unknown), and adjuvant therapy (yes/no/unknown).

When stratifying patients by tumor location, a statistically significant worse CRC‐specific and overall‐survival was observed for patients with *KRAS*_*mut*_ + pMMR cancers and *BRAF*_*mut*_ + pMMR cancers located in the colon or rectum compared to patients with all‐wild‐type + pMMR cancers in the colon or rectum (Table 2). Moreover, patients with other + pMMR cancers located in the colon had a statistically significant worse survival compared to patients with all‐wild‐type + pMMR cancers located in the colon. Patients with *PIK3CA*_*mut*_ + pMMR cancer in the rectum showed a borderline statistically significant (possibly because of low power) worse overall survival (HR_overall_ 1.62; 95% CI 0.97–2.73) compared to patients with all‐wild‐type + pMMR rectal cancer. No statistically significant survival differences were observed for any of the mutational subgroups in patients with cancers located in the rectosigmoid (Table 2).

Next, we stratified CRC patients by pTNM stage to assess the disease stage‐dependent prognostic value of the mutational subgroups (Table S6). In pTNM stage I, similar associations were observed for CRC‐specific survival of the mutational subgroups, whereas no statistically significant associations were observed for overall survival. Compared to patients with all‐wild‐type + pMMR CRC, only patients with *KRAS*_*mut*_ + *pMMR* CRC (HR 1.52; 95% CI 1.07–2.15) had a significantly worse CRC‐specific survival in pTNM Stage II. For pTNM stages III and IV, patients with *BRAF*_*mut*_ + pMMR CRC had a significantly worse CRC‐specific and overall survival compared to patients with all‐wild‐type + pMMR CRC. Moreover, patients with other + pMMR CRC had a significantly worse overall survival (HR 1.49; 95% CI 1.05–2.12) in pTNM stage III. Patients with *KRAS*_*mut*_ + pMMR CRC had a significantly worse CRC‐specific (HR 1.37; 95% CI 1.02–1.85) and overall survival (HR 1.30; 95% CI 0.98–1.73) compared to patients with all‐wild‐type + pMMR CRC in pTNM Stage IV. Lastly, patients with other + pMMR CRC had a (borderline) significantly worse overall survival in pTNM Stage IV, whereas patients with other + dMMR CRC had a significantly better CRC‐specific and overall survival in pTNM Stage IV (Table S6).

### 3.4 Relationship between mutational subgroups and Warburg‐subtypes

After excluding patients with missing protein expression data on LDHA, GLUT1, MCT4, PKM2, p53, or PTEN (*n* = 76), 2268 CRC patients with information on Warburg‐subtype and mutational status were available for analyses (Warburg‐low: *n* = 646, 28.5%; Warburg‐moderate: *n* = 820, 36.2%; Warburg‐high: *n* = 802, 35.4%).

A cross‐tabulation of the mutational subgroups by Warburg‐subtypes for all CRC as well as for colon, rectosigmoid and rectal cancers separately is shown in Table 3. All‐wild‐type + pMMR, *PIK3CA*_*mut*_ + pMMR, and other + pMMR CRC were more frequently classified as Warburg‐low. *BRAF*_*mut*_ and other + dMMR CRC were more frequently classified as Warburg‐high. *KRAS*_*mut*_ + pMMR CRC were more frequently classified as Warburg‐moderate or Warburg‐high. Stratifying on tumor location showed similar results, except for cancers located in the rectum, where *PIK3CA*_*mut*_ + pMMR cancers were more frequently classified as Warburg‐high. When stratifying on pTNM stage (Table S7) similar results were observed.

**TABLE 3:** Frequencies of the mutational subgroups, stratified on tumor location (colon, rectosigmoid, rectum) and Warburg‐subtype (Warburg‐low, −moderate, −high)

	Total *n* (%)	Warburg‐low *n* (%)	Warburg‐moderate *n* (%)	Warburg‐high *n* (%)
Colorectal				
All‐wild‐type+pMMR	827 (36.5)	285 (44.1)	300 (36.6)	242 (30.2)
*KRAS*_*mut*_ + pMMR	554 (24.4)	128 (19.8)	226 (27.6)	200 (24.9)
*KRAS*_*mut*_ + *PIK3CA*_*mut*_ + pMMR	168 (7.4)	48 (7.4)	69 (8.4)	51 (6.4)
*PIK3CA*_*mut*_ + pMMR	118 (5.2)	43 (6.7)	36 (4.4)	39 (4.9)
*BRAF*_*mut*_ + pMMR	144 (6.4)	24 (3.7)	38 (4.6)	82 (10.2)
*BRAF*_*mut*_ + dMMR	132 (5.8)	32 (5.0)	39 (4.8)	61 (7.6)
Other + pMMR	211 (9.3)	63 (9.8)	75 (9.2)	73 (9.1)
Other + dMMR	114 (5.0)	23 (3.6)	37 (4.5)	54 (6.7)
Colon				
All‐wild‐type + pMMR	501 (31.2)	159 (37.1)	187 (32.2)	155 (25.9)
*KRAS*_*mut*_ + pMMR	374 (23.3)	81 (18.9)	154 (26.5)	139 (23.2)
*KRAS*_*mut*_ + *PIK3CA*_*mut*_ + pMMR	129 (8.0)	36 (8.4)	53 (9.1)	40 (6.7)
*PIK3CA*_*mut*_ + pMMR	88 (5.5)	34 (7.9)	29 (5.0)	25 (4.2)
*BRAF*_*mut*_ + pMMR	129 (8.0)	23 (5.4)	33 (5.7)	73 (12.2)
*BRAF*_*mut*_ + dMMR	130 (8.1)	31 (7.2)	38 (6.5)	61 (10.2)
Other + pMMR	146 (9.1)	43 (10.0)	51 (8.8)	52 (8.7)
Other + dMMR	111 (6.9)	22 (5.1)	36 (6.2)	53 (8.9)
Rectosigmoid				
All‐wild‐type + pMMR	112 (50.2)	51 (66.2)	35 (45.5)	26 (37.7)
*KRAS*_*mut*_ + pMMR	69 (30.9)	13 (16.9)	27 (35.1)	29 (42.0)
*KRAS*_*mut*_ + *PIK3CA*_*mut*_ + pMMR	12 (5.4)	3 (3.9)	5 (6.5)	4 (5.8)
*PIK3CA*_*mut*_ + pMMR	8 (3.6)	4 (5.2)	1 (1.3)	3 (4.4)
*BRAF*_*mut*_ + pMMR	3 (1.4)	1 (1.3)	–	2 (2.9)
*BRAF*_*mut*_ + dMMR	1 (0.5)	1 (1.3)	–	–
Other + pMMR	18 (8.1)	4 (5.2)	9 (11.7)	5 (7.3)
Other + dMMR	–	–	–	–
Rectum				
All‐wild‐type + pMMR	214 (49.0)	75 (53.6)	78 (48.2)	61 (45.2)
*KRAS*_*mut*_ + pMMR	111 (25.4)	34 (24.3)	45 (27.8)	32 (23.7)
*KRAS*_*mut*_ + *PIK3CA*_*mut*_ + pMMR	27 (6.2)	9 (6.4)	11 (6.8)	7 (5.2)
*PIK3CA*_*mut*_ + pMMR	22 (5.0)	5 (3.6)	6 (3.7)	11 (8.2)
*BRAF*_*mut*_ + pMMR	12 (2.8)	–	5 (3.1)	7 (5.2)
*BRAF*_*mut*_ + dMMR	1 (0.2)	–	1 (0.6)	–
Other + pMMR	47 (10.8)	16 (11.4)	15 (9.3)	16 (11.9)
Other + dMMR	3 (0.7)	1 (0.7)	1 (0.6)	1 (0.7)

### 3.5 Survival of Warburg‐subtypes within mutational subgroups

Univariable Kaplan–Meier curves showed no statistically significant survival differences between Warburg‐subtypes within any of the mutational subgroups (Figure S1).

Multivariable‐adjusted analyses showed that, compared to patients with Warburg‐low CRC, patients with Warburg‐high CRC had a (borderline) statistically significant worse CRC‐specific (HR 1.16; 95% CI 0.98–1.37) and overall survival (HR 1.20; 95% CI 1.05–1.36) (Table 4). Further analyses according to mutational subgroups showed no statistically significant associations with survival across Warburg‐subtypes within any of the mutational subgroups. Worse, though not statistically significant, CRC‐specific and overall survival was observed for the Warburg‐high subtype as compared to the Warburg‐low subtype in patients with *KRAS*_*mut*_ + pMMR CRC (HR_CRC‐specific_ 1.31; 95% CI 0.94–1.84 and HR_overall_ 1.27; 95% CI 0.96–1.68), *BRAF*_*mut*_ + pMMR CRC (HR_CRC‐specific_ 1.42; 95% CI 0.74–2.71 and HR_overall_ 1.13; 95% CI 0.65–1.95), and *BRAF*_*mut*_ + dMMR CRC (HR_CRC‐specific_ 1.41; 95% CI 0.60–3.31 and HR_overall_ 1.54; 95% CI 0.83–2.87) (Table 4). In contrast, the Warburg‐high subtype was not associated with CRC‐specific or overall survival in patients with all‐wild‐type + pMMR CRC, *KRAS*_*mut*_ + *PIK3CA*_*mut*_ + pMMR CRC, and *PIK3CA*_*mut*_ + pMMR CRC (Table 4).

**TABLE 4:** Univariable and multivariable‐adjusted hazard ratios for associations between Warburg‐subtypes and survival of colorectal cancer patients within the Netherlands Cohort Study (NLCS, 1986‐2006) in mutational subgroups

	*N*	CRC‐specific survival			Overall survival		
		CRC deaths *n* (%)	HR (95% CI)		Deaths *n* (%)	HR (95% CI)	
			Univariable	Multivariable‐adjusted^*^		Univariable	Multivariable‐adjusted^*^
Total							
Warburg‐low	646	241 (37.3)	1.00 (ref)	1.00 (ref)	393 (60.8)	1.00 (ref)	1.00 (ref)
Warburg‐moderate	820	343 (41.8)	1.15 (0.98‐1.36)	1.07 (0.91‐1.26)	526 (64.1)	1.09 (0.96‐1.24)	1.05 (0.92‐1.20)
Warburg‐high	802	346 (43.1)	1.26 (1.07‐1.49)	1.16 (0.98‐1.37)	550 (68.6)	1.25 (1.10‐1.43)	1.20 (1.05‐1.36)
All‐wild‐type+pMMR							
Warburg‐low	285	97 (34.0)	1.00 (ref)	1.00 (ref)	173 (60.7)	1.00 (ref)	1.00 (ref)
Warburg‐moderate	300	112 (37.3)	1.07 (0.82‐1.41)	0.99 (0.75‐1.31)	177 (59.0)	0.95 (0.77‐1.17)	0.94 (0.76‐1.16)
Warburg‐high	242	93 (38.4)	1.22 (0.92‐1.62)	0.98 (0.72‐1.32)	163 (67.4)	1.22 (0.98‐1.51)	1.10 (0.88‐1.38)
*KRAS*_*mut*_+ pMMR							
Warburg‐low	128	55 (43.0)	1.00 (ref)	1.00 (ref)	81 (63.3)	1.00 (ref)	1.00 (ref)
Warburg‐moderate	226	104 (46.0)	1.08 (0.78‐1.50)	1.06 (0.76‐1.48)	149 (65.9)	1.06 (0.81‐1.39)	1.00 (0.76‐1.32)
Warburg‐high	200	105 (52.5)	1.39 (1.00‐1.92)	1.31 (0.94‐1.84)	147 (73.5)	1.35 (1.03‐1.78)	1.27 (0.96‐1.68)
*KRAS*_*mut*_+ *PIK3CA*_*mut*_ + pMMR							
Warburg‐low	48	22 (45.8)	1.00 (ref)	1.00 (ref)	30 (62.5)	1.00 (ref)	1.00 (ref)
Warburg‐moderate	69	31 (44.9)	1.04 (0.60‐1.80)	1.25 (0.70‐2.23)	46 (66.7)	1.11 (0.70‐1.76)	1.30 (0.81‐2.11)
Warburg‐high	51	21 (41.2)	0.95 (0.52‐1.72)	0.95 (0.48‐1.88)	32 (62.7)	1.03 (0.63‐1.70)	1.07 (0.62‐1.86)
*PIK3CA*_*mut*_ + pMMR							
Warburg‐low	43	16 (37.2)	1.00 (ref)	1.00 (ref)	22 (51.2)	1.00 (ref)	1.00 (ref)
Warburg‐moderate	36	14 (38.9)	1.14 (0.56‐2.34)	0.63 (0.28‐1.43)	25 (69.4)	1.57 (0.88‐2.79)	1.22 (0.65‐2.27)
Warburg‐high	39	12 (30.8)	0.88 (0.41‐1.85)	0.92 (0.39‐2.17)	27 (69.2)	1.50 (0.86‐2.64)	1.59 (0.85‐2.97)
*BRAF*_*mut*_+ pMMR							
Warburg‐low	24	12 (50.0)	1.00 (ref)	1.00 (ref)	18 (75.0)	1.00 (ref)	1.00 (ref)
Warburg‐moderate	38	27 (71.1)	1.49 (0.76‐2.95)	1.22 (0.59‐2.50)	31 (81.6)	1.15 (0.64‐2.05)	0.99 (0.53‐1.83)
Warburg‐high	82	51 (62.2)	1.16 (0.62‐2.18)	1.42 (0.74‐2.71)	61 (74.4)	0.92 (0.54‐1.55)	1.13 (0.65‐1.95)
*BRAF*_*mut*_+ dMMR							
Warburg‐low	32	10 (31.3)	1.00 (ref)	1.00 (ref)	16 (50.0)	1.00 (ref)	1.00 (ref)
Warburg‐moderate	39	13 (33.3)	1.03 (0.45‐2.35)	1.46 (0.61‐3.54)	24 (61.5)	1.18 (0.63‐2.22)	1.52 (0.78‐2.96)
Warburg‐high	61	21 (34.4)	1.13 (0.53‐2.40)	1.41 (0.60‐3.31)	42 (68.9)	1.44 (0.81‐2.56)	1.54 (0.83‐2.87)
Other + pMMR							
Warburg‐low	63	28 (44.4)	1.00 (ref)	1.00 (ref)	42 (66.7)	1.00 (ref)	1.00 (ref)
Warburg‐moderate	75	33 (44.0)	1.06 (0.64‐1.75)	1.18 (0.70‐1.99)	54 (72.0)	1.19 (0.79‐1.78)	1.30 (0.85‐1.97)
Warburg‐high	73	30 (41.1)	0.91 (0.55‐1.53)	0.95 (0.55‐1.64)	48 (65.8)	0.98 (0.65‐1.48)	1.02 (0.66‐1.58)
Other + dMMR							
Warburg‐low	23	1 (4.3)	1.00 (ref)	1.00 (ref)	11 (47.8)	1.00 (ref)	1.00 (ref)
Warburg‐moderate	37	9 (24.3)	7.18 (0.91‐56.75)	5.03 (0.59‐42.62)	20 (54.1)	1.55 (0.74‐3.24)	1.37 (0.63‐2.99)
Warburg‐high	54	13 (24.1)	7.09 (0.93‐54.27)	8.13 (0.93‐71.34)	30 (55.6)	1.61 (0.80‐3.21)	1.65 (0.78‐3.50)

Abbreviations: CI, confidence interval; CRC, colorectal cancer; HR, hazard ratio; pMMR, mismatch repair proficient; dMMR, mismatch repair deficient.

* Adjusted for age at diagnosis (years), sex (men/women), tumor location (colon/rectosigmoid/rectum), pTNM stage (I/II/III/IV/unknown), differentiation grade (well/moderate/poor/unknown), and adjuvant therapy (yes/no/unknown).

### 3.6 Sensitivity analyses

In sensitivity analyses, excluding CRC patients with unknown pTNM stage, differentiation grade, or missing information with respect to adjuvant therapy yielded similar results, except for a statistically significant worse overall survival for patients with *KRAS*_*mut*_ + pMMR CRC in pTNM stage III (HR 1.32; 95% CI 1.02–1.71), and a borderline statistically significant difference in CRC‐specific survival for patients with *KRAS*_*mut*_ + pMMR CRC in pTNM stage IV (HR 1.30; 95% CI 0.96–1.78) (*data not shown*). Furthermore, a statistically significant positive association was found between the Warburg‐high subtype and overall‐ and CRC‐specific survival (HR 1.49; 95%CI 1.04–2.13 and HR 1.44; 95%CI 1.07–1.94, respectively) in patients with *KRAS*_*mut*_ + pMMR CRC (*data not shown*).

## 4 DISCUSSION

In this large population‐based series of CRC patients, we have investigated the association between mutational subgroups and patient survival. Moreover, we investigated the relationship between previously identified Warburg‐subtypes^23^ and survival within these mutational subgroups to examine whether Warburg‐subtypes provide additional prognostic value.

CRC patients were classified into eight mutually exclusive mutational subgroups, based on the presence of somatic mutations in *RAS* (*KRAS*, *NRAS*, *HRAS*), *BRAF*, *PIK3CA*, *MET*, as well as, patients' mismatch repair (MMR) status: (1) All‐wild‐type + pMMR, (2) *KRAS*_*mut*_ + pMMR, (3) *KRAS*_*mut*_ + *PIK3CA*_*mut*_ + pMMR, (4) *PIK3CA*_*mut*_ + pMMR, (5) *BRAF*_*mut*_ + pMMR, (6) *BRAF*_*mut*_ + dMMR, (7) other + pMMR, and (8) other + dMMR. The other + dMMR subgroup largely consisted of patients with all‐wild‐type CRC or patients with mutations in *BRAF* and/or *PIK3CA*, whereas, the other + pMMR subgroup mainly consisted of patients with mutations in *RAS (NRAS*, *KRAS)* and/or *MET*.

We found important survival differences across mutational subgroups, independent of known prognostic factors like pTNM stage. Compared to patients with all‐wild‐type + pMMR CRC, patients with *KRAS*_mut_ + pMMR, *KRAS*_*mut*_ + *PIK3CA*_*mut*_ + pMMR, *BRAF*_*mut*_ + pMMR or other + pMMR CRC had a worse survival. Patients with *BRAF*_*mut*_ + pMMR CRC had the poorest survival, whereas patients with other + dMMR CRC had the most favorable survival. In relation to our previously defined Warburg‐subtypes, our results suggest that *BRAF*_*mut*_, *KRAS*_*mut*_ + pMMR, and other + dMMR CRC may be related to the Warburg‐high subtype. Lastly, we did not observe statistically significant survival differences for the Warburg‐subtypes within mutational subgroups.

Mutation frequencies of *RAS (KRAS*, *NRAS*, *HRAS)*, *BRAF*, *PIK3CA*, and *MET*, as well as the frequency of dMMR in this study are similar to those reported previously^33^, ^34^, ^35^ and those described in the COSMIC database.^17^, ^36^ Moreover, our results confirm previous reports that *BRAF* mutations occur frequently in dMMR CRC, whereas co‐existence of *KRAS* mutations and *BRAF* mutations or dMMR are rare.^37^, ^38^ In addition, our study confirms that *PIK3CA* mutations often co‐exist with other mutations, and especially with *KRAS* mutations, as reported previously.^34^

In the present study, we found that compared to patients with all‐wild‐type + pMMR CRC, patients with *KRAS*_mut_ + pMMR CRC had a poor survival. No significant association with survival was observed for patients with *PIK3CA*_*mut*_ + pMMR CRC whereas patients with *KRAS*_*mut*_ + *PIK3CA*_*mut*_ + pMMR had a worse CRC‐specific survival, suggesting that *KRAS* mutations may drive the worse survival observed for this subgroup. The survival of patients with *BRAF‐*mutated CRC was highly dependent on MMR status. Patients with *BRAF*_*mut*_ + pMMR CRC had the poorest survival, whereas no difference in survival was found for patients with *BRAF*_*mut*_ + dMMR CRC. These results suggest that dMMR may ‘override’ the negative prognostic potential of *BRAF* mutations. In addition, our results indicate that patients with other + pMMR CRC have a poor survival. The most favorable survival was observed for patients with other + dMMR CRC, again highlighting the favorable prognostic value of dMMR.

Many studies have investigated the prognostic value of MMR status, *KRAS‐*, *BRAF‐*, or *PIK3CA*‐mutations in CRC in the past. However, most studies did not evaluate these mutations exclusively (e.g., patients with a *KRAS*‐mutant or *KRAS* wild‐type cancer may have had another mutation in a different gene),^39^ which could have potentially diluted their results. Studies assessing the prognostic value of CRC subgroups, based on combinations of frequently occurring mutations (*RAS*, *BRAF*, *PIK3CA)* and/or MMR status, are very limited and rarely evaluate all markers at the same time.^40^, ^41^, ^42^, ^43^

MSI status is most consistently associated with CRC survival.^40^ It has been shown that patients with MSI high (MSI‐H) CRC have a better overall survival compared to patients with microsatellite stable (MSS) CRC.^40^ Mutations in *BRAF* have also consistently been associated with poor survival in CRC.^44^, ^45^ In contrast, the prognostic significance of mutations in *KRAS* and/or *PIK3CA* is unclear, as results of previous studies are inconsistent.^42^, ^46^, ^47^ More recently, several studies have investigated the association between combinations of markers and CRC survival. Various studies have reported on the association between MMR status in combination with *BRAF* or *KRAS* mutations and CRC survival. In line with our results, it has been shown that the adverse effect of mutant *BRAF* on survival is limited to MSS CRC.^20^, ^22^, ^43^, ^48^ In addition, a poorer survival was reported for patients with MSS and a *KRAS* mutation, compared to the reference group (i.e., MSS, *BRAF*_wild‐type_, and *KRAS*_wild‐type_).^40^, ^43^, ^49^ These and our results suggest a complex interplay between these markers and highlight the importance of evaluating multiple markers at the same time.

Even though future studies—with higher numbers of CRC patients within each of the subgroups—are necessary to validate our findings and to investigate the biological basis for the observed differences in subgroup‐specific survival, a potential mechanism may be the involvement of the Warburg‐effect.

It has been suggested that mutations in *RAS (KRAS*, *NRAS*, *HRAS)*, *BRAF*, and *PIK3CA* promote the Warburg‐effect through activation of the *PI3K/AKT/mTOR* and *RAS/RAF/MEK/ERK* oncogenic pathways.^12^, ^13^, ^14^ We have previously shown that patients with Warburg‐high CRC (i.e., a high probability of the presence of the Warburg‐effect) had a worse survival compared to patients with Warburg‐low CRC, especially in patients with rectal cancers or pTNM stage III CRC.^23^ To our knowledge, this is the first study to investigate the relationship between mutational subgroups and these previously defined Warburg‐subtypes, and to examine whether Warburg‐subtypes provide additional prognostic value within mutational subgroups in CRC. The results of the present study suggest that *BRAF*_*mut*_, *KRAS*_*mut*_ + pMMR, and other + dMMR subgroups may be related to the Warburg‐high subtype in cancers located in the colon and rectum. In addition, the *PIK3CA*_*mut*_ + pMMR subgroup seems to be related to the Warburg‐high subtype in cancers located in the rectum. We did not find statistically significant survival differences across Warburg‐subtypes within mutational subgroups. This might be due to limited statistical power when subclassifying based on mutational subgroups and Warburg‐subtypes despite investigating a very large cohort of CRC. Similarly, associations may be concealed overall as we did not have enough power to stratify our analyses on tumor location or pTNM stage.

The main strengths of this study include the use of a large population‐based series of incident CRC patients, the nearly complete follow‐up, the fact that patients were mainly treated with surgery, and the availability of DNA and tumor material for a large number of CRC patients. Our study has some limitations. First, the ColoCarta panel that was used includes assays for most known *KRAS* (99%) and *BRAF* (98%) mutations, but only 78% of known *PIK3CA* mutations.^35^ Second, we determined MMR status as a proxy for MSI status, which might have led to misclassification of some CRC patients. However, it has been described that IHC analysis of MLH1 and MSH2 expression is a reliable method for the detection of the vast majority of patients with MSI CRC.^50^ Third, our study did not have a validation cohort available to confirm the observed associations. Fourth, we made no adjustments for multiple testing which may have potentially resulted in change findings. Therefore, our results should be interpreted with caution, and validation of the current findings is required. Fifth, we did not have detailed clinical information available regarding the exact type, duration or dosage of treatment. Lastly, other limitations with regard to Warburg‐subtyping have been described previously.^23^

## 5 CONCLUSION

In this large, population‐based series of CRC patients, we have shown that mutational subgroups, based on the observed mutation frequencies of *RAS (KRAS*, *NRAS*, *HRAS)*, *BRAF*, *PIK3CA*, and *MET*, as well as patients' MMR status, are associated with important differences in survival. Our results suggest that *BRAF*_*mut*_, *KRAS*_*mut*_ + pMMR, and other + dMMR subgroups may be related to the Warburg‐high subtype in cancers located in the colon or rectum. However, no statistically significant survival differences were observed for the Warburg‐subtypes within mutational subgroups. All in all, our results highlight the prognostic value of mutational subgroups in CRC. In the future, CRC‐subtyping based on mutational subgroups may be used for risk stratification, the design of (combined) targeted therapies, and to improve therapeutic outcomes of CRC patients. Future, larger‐scale prospective studies or pooled studies are necessary to validate our findings, to further explore the potential prognostic value of Warburg‐subtypes, and to examine the potential clinical utility of CRC subtyping based on mutational subgroups.

## ETHICS STATEMENT

The NLCS was approved by institutional review boards from Maastricht University and the Netherlands Organization for Applied Scientific Research. Ethical approval was obtained from Medical Ethical Committee of Maastricht University Medical Center+. By completing and returning the questionnaire, participants agreed to participate in the study.

## AUTHOR CONTRIBUTIONS

Conceptualization: Piet A. van den Brandt, Heike I. Grabsch, Colinda C. J. M. Simons, Josien C.A. Jenniskens, Kelly Offermans; Methodology: Piet A. van den Brandt; Data acquisition: Piet A. van den Brandt, Leo J. Schouten; Formal analysis and investigation: Kelly Offermans; Writing—original draft preparation: Piet A. van den Brandt, Heike I. Grabsch, Josien C.A. Jenniskens, Kelly Offermans; Writing—review and editing: Piet A. van den Brandt, Heike I. Grabsch, Colinda C. J. M. Simons, Matty P. Weijenberg, Leo J. Schouten, Kim M. Smits, Iryna Samarska, Gregorio E. Fazzi, Jaleesa R. M. van der Meer, Josien C.A. Jenniskens; Funding acquisition: Piet A. van den Brandt, Heike I. Grabsch; Supervision: Piet A. van den Brandt, Heike I. Grabsch.

## FUNDING INFORMATION

This project was funded by The Dutch Cancer Society (KWF 11044 to P.A. van den Brandt).

## CONFLICT OF INTEREST

HG has received honoraria from Astra Zeneca and BMS for scientific advisory board activities not related to the current study. The remaining authors have no conflicts of interest to declare.

## Supporting information


Table S1–S7
Fig S1

## Data Availability

The anonymized data that are minimally required to replicate the outcomes of the study are available from the corresponding author upon reasonable request.
