# Supplementary material for: Association between mutational subgroups, Warburg‐subtypes, and survival in patients with colorectal cancer
Source: Cancer Med. 2022 Jul 3;12(2):1137–56. doi: 10.1002/cam4.4968 (PMC9883416; doi:10.1002/cam4.4968)
Supplement: Supplementary file 1 — Table S1–S7 Fig S1 [file CAM4-12-1137-s001.docx]

**Supplementary Table S1** – Details primary antibodies and staining protocols.

| **Antibody** | **Clone** | **Source** | **Dilution** | **Staining procedure** | **Antigen retrieval** | **Incubation time** | **Visualisation system** | **Chromogen** |
| --- | --- | --- | --- | --- | --- | --- | --- | --- |
| Pan-CK | AE1/AE3 | DAKO (GA053) | RTU | DAKO Autostainer^a^ | PT high^b^ | 10 minutes | EnVision FLEX^f^ | DAB |
| MLH1 | ES05 | DAKO (M3640) | RTU | DAKO Autostainer^a^ | PT high^b^ | 40 minutes | EnVision FLEX^f^ | DAB |
| MSH2 | FE11 | DAKO (M3639) | RTU | DAKO Autostainer^a^ | PT high^b^ | 40 minutes | EnVision FLEX^f^ | DAB |
| TP53 | DO-7 | DAKO (M7001) | RTU | DAKO Autostainer^a^ | PT high^b^ | 20 minutes | EnVision FLEX^f^ | DAB |
| PTEN | 6H2.1 | DAKO (M3627) | 1:100 | DAKO Autostainer^a^ | PT high^b^ | 20 minutes | EnVision FLEX^f^ | DAB |
| GLUT1 | - | TFS (RB-9052-P1) | 1:200 | DAKO Autostainer^a^ | PT low^c^ | 20 minutes | EnVision FLEX^f^ | DAB |
| LDHA | E-9 | SCBT (sc-137243) | 1:800 | Manual | HIER high^d^ | Overnight, 4°C | REAL EnVision^g^ | DAB |
| MCT4 | D-1 | SCBT (sc-376140) | 1:100 | Manual | HIER high^d^ | Overnight, 4°C | REAL EnVision^g^ | DAB |
| PKM2 | C-11 | SCBT (sc-365684) | 1:100 | Manual | HIER low^e^ | 1hr, 37°C | LSAB2 Kit/HRP^h^ | DAB |
| ^a^DAKO Autostainer Link 48  ^b^High pH retrieval (K8004) for 20 minutes on the Dako PT link (Agilent Technologies)  ^c^Low pH retrieval (K8005) for 20 minutes on the Dako PT link (Agilent Technologies)  ^d^Heat-induced antigen retrieval using a solution of Tris/EDTA (pH 9.0)  ^e^Heat-induced antigen retrieval using a solution of sodium citrate (pH 6.0)  ^f^EnVision FLEX Visualization Kit (K8008, DAKO)  ^g^REAL EnVision Detection System (K5007, DAKO)  ^h^Universal LSAB2 kit/HRP (K0675, Agilent)  TFS, Thermo Fisher Scientific; SBCT, Santa Cruz Biotechnology; RTU, ready-to-use; DAB, 3,3’-diaminobenzide; | | | | | | | | |

**Supplementary Table S2** – Scoring protocols and kappa values with 95% confidence intervals for inter- and intra-observer agreement of the six proteins incorporated in the Warburg-subtypes

|  | | **P53** | **PTEN** | **GLUT1** | **LDHA** | **MCT4** | **PKM2** |
| --- | --- | --- | --- | --- | --- | --- | --- |
|  | |  |  |  |  |  |  |
| **Localisation^a^** | | Nucleus | Cytoplasm^b^ | Membrane | Cytoplasm | Membrane | Cytoplasm |
|  | |  |  |  |  |  |  |
|  | |  |  |  |  |  |  |
| **Scoring protocol** | |  |  |  |  |  |  |
|  | |  |  |  |  |  |  |
| **Low** | |  |  |  |  |  |  |
|  | Category 1 | (1) negative | (1) negative | (1) negative | (1) negative/weak | (1) negative | (1) negative/weak |
|  | Category 2 | (2) 1-10% positive |  | (2) 1-10% positive |  | (2) 1-10% positive |  |
| **Moderate** | |  |  |  |  |  |  |
|  | Category 2 |  | (2) weak |  | (2) 1-50% strong positive |  | (2) moderate positive |
|  | Category 3 | (3) 11-50% positive | (3) moderate | (3) 11-50% positive |  | (3) 11-50% positive | (3) 1-50% strong positive |
| **High** | |  |  |  |  |  |  |
|  | Category 3 |  |  |  | (3) >50% strong positive |  |  |
|  | Category 4 | (4) 51-90% positive | (4) strong | (4) >50% positive |  | (4) >50% positive | (4) >50% strong positive |
|  | Category 5 | (5) >90% positive |  |  |  |  |  |
|  | |  |  |  |  |  |  |
|  | |  |  |  |  |  |  |
| **Scoring agreement** | |  |  |  |  |  |  |
|  | |  |  |  |  |  |  |
| **Inter-observer agreement^c^** | | **ᴋ (95%-CI)** | **ᴋ (95%-CI)** | **ᴋ (95%-CI)** | **ᴋ (95%-CI)** | **ᴋ (95%-CI)** | **ᴋ (95%-CI)** |
| Final score^d^ vs pathologist | |  |  |  |  |  |  |
|  | Weighted kappa^e^ | 0.75 (0.72-0.79) | 0.58 (0.53-0.62) | 0.71 (0.67-0.74) | 0.65 (0.60-0.69) | 0.74 (0.71-0.77) | 0.65 (0.61-0.69) |
|  | Non-weighted kappa | 0.63 (0.58-0.68) | 0.47 (0.41-0.52) | 0.61 (0.57-0.66) | 0.59 (0.54-0.64) | 0.63 (0.59-0.68) | 0.56 (0.51-0.60) |
|  | |  |  |  |  |  |  |
| **Intra-observer agreement^c, f^** | |  |  |  |  |  |  |
| Non-pathologist assessor 1 | |  |  |  |  |  |  |
|  | Weighted kappa^e^ | 0.83 (0.80-0.86) | 0.69 (0.65-0.74) | 0.82 (0.79-0.85) | 0.78 (0.74-0.82) | 0.86 (0.83-0.88) | 0.70 (0.67-0.74) |
|  | Non-weighted kappa | 0.73 (0.69-0.77) | 0.63 (0.58-0.69) | 0.75 (0.72-0.79) | 0.76 (0.71-0.80) | 0.79 (0.75-0.82) | 0.58 (0.53-0.62) |
| Non-pathologist assessor 2 | |  |  |  |  |  |  |
|  | Weighted kappa^e^ | 0.87 (0.84-0.90) | 0.69 (0.64-0.74) | 0.75 (0.72-0.78) | 0.77 (0.73-0.81) | 0.83 (0.81-0.86) | 0.72 (0.68-0.75) |
|  | Non-weighted kappa | 0.80 (0.76-0.84) | 0.65 (0.60-0.70) | 0.65 (0.60-0.69) | 0.73 (0.69-0.78) | 0.75 (0.71-0.79) | 0.62 (0.58-0.67) |
|  | |  |  |  |  |  |  |
| ^a^Only immunoreactivity in reported cellular localization was considered positive staining.  ^b^For PTEN scoring, staining intensity of tumour cells was compared with that of stromal cells: (1) no PTEN staining in the tumour cells; (2) staining intensity in the tumour cells weaker than in the stromal cells; (3) similar staining intensity in tumour and stromal cells; (4) staining intensity in the tumour cells stronger than in the stromal cells.  ^c^Based on a random 10% of TMA sections  ^d^The final score is based on at least two non-pathologists, with discrepancies replaced by a consensus score or pathologist’s score.  ^e^Weight of 0.5 for adjacent categories and 0 for non-adjacent categories.  ^f^10% of TMA sections were scored for a second time after at least 2 months. | | | | | | | |

**Supplementary Table** **S3** – ColoCarta panel genes and mutations

| **Gene** | **Assay** | **Mutation** |
| --- | --- | --- |
|  |  |  |
| *BRAF* | 15/16 | *V600E/K/L/M/R* |
|  | 9 | *D594G/V* |
|  |  |  |
| *KRAS* | 1 | *G12A/D/V* |
|  | 2 | *G12C/R/S* |
|  | 4 | *G13D/V* |
|  | 5 | *A59T* |
|  | 7 | *Q61L/P/R* |
|  | 8 | *Q61H_A/H_G* |
|  |  |  |
| *PIK3CA* | 1 | *R88Q* |
|  | 3 | *C420R* |
|  | 5 | *E542K* |
|  | 6 | *E545K* |
|  | 7 | *Q546K* |
|  | 8 | *H701P* |
|  | 9 | *H1047L/R* |
|  |  |  |
| *NRAS* | 1 | *G12A/D/V* |
|  | 2 | *G12C/R/S* |
|  | 3 | *G13A/D/V* |
|  | 4 | *G13C/R/S* |
|  | 7 | *Q61H* |
|  | 8 | *Q61E/K* |
|  |  |  |
| *HRAS* | 6 | *Q61L/P/R* |
|  |  |  |
| *MET* | 1 | *R970C* |
|  | 2 | *T992I* |
|  |  |  |

**Supplementary Table S4** – Mutational subgroups, based on the observed frequencies of (combinations of) tumour markers (i.e. KRAS, BRAF, PIK3CA, NRAS, MET, MMR).

| **Mutational subgroups** | **KRAS** | **BRAF** | **PIK3CA** | **NRAS** | **MET** | **MMR** |
| --- | --- | --- | --- | --- | --- | --- |
| **All-wild-type+pMMR** | wild-type | wild-type | wild-type | wild-type | wild-type | proficient |
| ***KRAS­_mut_+*pMMR** | mutant | wild-type | wild-type | wild-type | wild-type | proficient |
| ***KRAS_mut_+PIK3CA_mut_+*pMMR** | mutant | wild-type | mutant | wild-type | wild-type | proficient |
| ***PIK3CA_mut_+*pMMR** | wild-type | wild-type | mutant | wild-type | wild-type | proficient |
| ***BRAF_mut_+*pMMR** | wild-type | mutant | wild-type | wild-type | wild-type | proficient |
| ***BRAF_mut_+*dMMR** | wild-type | mutant | wild-type | wild-type | wild-type | deficient |
| **Other+pMMR** | wild-type/mutant | wild-type/mutant | wild-type/mutant | wild-type/mutant | wild-type/mutant | proficient |
| **Other+dMMR** | wild-type/mutant | wild-type/mutant | wild-type/mutant | wild-type/mutant | wild-type/mutant | deficient |

**Supplementary Table S5 –** TNM classification of colorectal cancer, according to incidence year.

| Topography | 153.0-154.1 or C18-C20 | | |
| --- | --- | --- | --- |
| Histology | Epithelial cancers (M8010-8580) | | |
|  |  |  |  |
| **Incidence years** | **1988-2002** |  |  |
| **TNM versions** | **4.1-5** |  |  |
|  |  |  |  |
| Stage | T | N | M |
| I | 1-2 | 0/X | 0/X |
| II | 3-4 | 0/X | 0/X |
| III | Any T | 1-3 | 0/X |
| IV | Any T | Any N | 1 |
| X | X | 0/X | 0/X |
|  |  |  |  |
|  |  |  |  |
| **Incidence years** | **2003-2009** |  |  |
| **TNM versions** | **6** |  |  |
|  |  |  |  |
| Stage | T | N | M |
| I | 1-2 | 0/X | 0/X |
| IIA | 3 | 0/X | 0/X |
| IIB | 4 | 0/X | 0/X |
| III | X | 1 | 0/X |
| IIIA | 1-2 | 1 | 0/X |
| IIIB | 3-4 | 1 | 0/X |
| IIIC | Any T | 2 | 0/X |
| IV | Any T | Any N | 1 |
| X | X | 0/X | 0/X |

**Supplementary Table S6 –** Univariable and multivariable-adjusted hazard ratios for associations between mutational subgroups and survival of colorectal cancer patients within the Netherlands Cohort Study (NLCS, 1986-2006), stratified on pTNM stage.

|  |  | N |  | **CRC-specific survival** | | |  | **Overall survival** | | |
| --- | --- | --- | --- | --- | --- | --- | --- | --- | --- | --- |
|  |  |  |  | CRC deaths (%) | HR (95% CI) | |  | Deaths (%) | HR (95% CI) | |
|  |  |  |  |  | Univariable | Multivariable-adjusted^a^ |  |  | Univariable | Multivariable-adjusted^a^ |
| **pTNM stage I** | |  |  |  |  |  |  |  |  |  |
|  | All-wild-type+pMMR | 194 |  | 27 (13.9) | 1.00 (ref) | 1.00 (ref) |  | 86 (44.3) | 1.00 (ref) | 1.00 (ref) |
|  | *KRAS_mut_+*pMMR | 119 |  | 29 (24.4) | 1.85 (1.09-3.12) | 2.06 (1.21-3.50) |  | 56 (47.1) | 1.13 (0.80-1.58) | 1.18 (0.84-1.66) |
|  | *KRAS_mut_+PIK3CA_mut_+*pMMR | 29 |  | 6 (20.7) | 1.63 (0.67-3.94) | 1.93 (0.79-4.73) |  | 16 (55.2) | 1.38 (0.81-2.36) | 1.31 (0.76-2.26) |
|  | *PIK3CA_mut_+*pMMR | 18 |  | 4 (22.2) | 1.72 (0.60-4.91) | 1.73 (0.60-5.01) |  | 9 (50.0) | 1.21 (0.61-2.41) | 1.40 (0.70-2.81) |
|  | *BRAF_mut_+*pMMR | 10 |  | 4 (40.0) | 3.52 (1.23-10.08) | 3.34 (1.12-9.94) |  | 7 (70.0) | 2.21 (1.02-4.77) | 1.44 (0.66-3.18) |
|  | *BRAF_mut_+*dMMR | 19 |  | 1 (5.3) | 0.41 (0.06-2.99) | 0.42 (0.06-3.21) |  | 8 (42.1) | 1.01 (0.49-2.09) | 0.91 (0.43-1.94) |
|  | Other+pMMR | 52 |  | 7 (13.5) | 1.00 (0.44-2.30) | 1.07 (0.46-2.46) |  | 22 (42.3) | 0.98 (0.61-1.56) | 1.08 (0.67-1.73) |
|  | Other+dMMR | 18 |  | 1 (5.6) | 0.37 (0.05-2.72) | 0.33 (0.04-2.49) |  | 7 (38.9) | 0.81 (0.38-1.76) | 0.67 (0.30-1.49) |
| **pTNM stage II** | |  |  |  |  |  |  |  |  |  |
|  | All-wild-type+pMMR | 305 |  | 68 (22.3) | 1.00 (ref) | 1.00 (ref) |  | 167 (54.8) | 1.00 (ref) | 1.00 (ref) |
|  | *KRAS_mut_+*pMMR | 185 |  | 60 (32.4) | 1.52 (1.08-2.16) | 1.52 (1.07-2.15) |  | 111 (60.0) | 1.17 (0.92-1.49) | 1.11 (0.87-1.41) |
|  | *KRAS_mut_+PIK3CA_mut_+*pMMR | 74 |  | 21 (28.4) | 1.29 (0.79-2.11) | 1.25 (0.76-2.04) |  | 39 (52.7) | 0.97 (0.69-1.38) | 0.92 (0.65-1.31) |
|  | *PIK3CA_mut_+*pMMR | 53 |  | 10 (18.9) | 0.81 (0.42-1.57) | 0.79 (0.40-1.54) |  | 30 (56.6) | 1.00 (0.68-1.47) | 0.98 (0.66-1.44) |
|  | *BRAF_mut_+*pMMR | 47 |  | 17 (36.2) | 1.64 (0.96-2.78) | 1.51 (0.87-2.62) |  | 25 (53.2) | 0.97 (0.64-1.48) | 0.90 (0.58-1.38) |
|  | *BRAF_mut_+*dMMR | 65 |  | 14 (21.5) | 0.95 (0.53-1.68) | 0.85 (0.47-1.55) |  | 36 (55.4) | 1.02 (0.71-1.46) | 0.87 (0.59-1.27) |
|  | Other+pMMR | 75 |  | 24 (32.0) | 1.48 (0.93-2.35) | 1.40 (0.87-2.24) |  | 46 (61.3) | 1.18 (0.85-1.63) | 1.15 (0.82-1.60) |
|  | Other+dMMR | 73 |  | 13 (17.8) | 0.79 (0.44-1.43) | 0.73 (0.40-1.35) |  | 38 (52.1) | 0.94 (0.66-1.33) | 0.89 (0.61-1.28) |
| **pTNM stage III** | |  |  |  |  |  |  |  |  |  |
|  | All-wild-type+pMMR | 220 |  | 116 (52.7) | 1.00 (ref) | 1.00 (ref) |  | 158 (71.8) | 1.00 (ref) | 1.00 (ref) |
|  | *KRAS_mut_+*pMMR | 157 |  | 78 (49.7) | 1.06 (0.80-1.42) | 1.14 (0.85-1.53) |  | 114 (72.6) | 1.15 (0.90-1.46) | 1.21 (0.94-1.55) |
|  | *KRAS_mut_+PIK3CA_mut_+*pMMR | 40 |  | 22 (55.0) | 1.10 (0.70-1.74) | 1.18 (0.75-1.87) |  | 27 (67.5) | 0.98 (0.65-1.48) | 1.06 (0.70-1.60) |
|  | *PIK3CA_mut_+*pMMR | 30 |  | 14 (46.7) | 0.89 (0.51-1.55) | 0.97 (0.55-1.69) |  | 20 (66.7) | 0.93 (0.58-1.48) | 1.04 (0.65-1.67) |
|  | *BRAF_mut_+*pMMR | 59 |  | 41 (69.5) | 2.06 (1.44-2.95) | 2.06 (1.41-3.02) |  | 49 (83.1) | 1.82 (1.32-2.51) | 1.76 (1.25-2.48) |
|  | *BRAF_mut_+*dMMR | 37 |  | 18 (48.6) | 1.14 (0.70-1.88) | 1.14 (0.67-1.95) |  | 27 (73.0) | 1.26 (0.84-1.89) | 1.16 (0.74-1.80) |
|  | Other+pMMR | 51 |  | 29 (56.9) | 1.30 (0.86-1.95) | 1.42 (0.94-2.14) |  | 40 (78.4) | 1.33 (0.94-1.89) | 1.49 (1.05-2.12) |
|  | Other+dMMR | 20 |  | 6 (30.0) | 0.56 (0.25-1.27) | 0.54 (0.23-1.26) |  | 12 (60.0) | 0.80 (0.44-1.44) | 0.78 (0.43-1.44) |
| **pTNM stage IV** | |  |  |  |  |  |  |  |  |  |
|  | All-wild-type+pMMR | 102 |  | 92 (90.2) | 1.00 (ref) | 1.00 (ref) |  | 102 (100.0) | 1.00 (ref) | 1.00 (ref) |
|  | *KRAS+*pMMR | 105 |  | 98 (93.3) | 1.25 (0.94-1.66) | 1.37 (1.02-1.85) |  | 104 (99.0) | 1.18 (0.90-1.56) | 1.30 (0.98-1.73) |
|  | *KRAS+PIK3CA+*pMMR | 27 |  | 26 (96.3) | 1.37 (0.88-2.11) | 1.36 (0.86-2.13) |  | 27 (100.0) | 1.28 (0.84-1.96) | 1.26 (0.81-1.95) |
|  | *PIK3CA+*pMMR | 18 |  | 15 (83.3) | 0.87 (0.50-1.51) | 1.09 (0.62-1.93) |  | 17 (94.4) | 0.84 (0.50-1.42) | 1.06 (0.62-1.81) |
|  | *BRAF+*pMMR | 27 |  | 25 (92.6) | 2.19 (1.41-3.42) | 1.97 (1.25-3.10) |  | 27 (100.0) | 2.14 (1.40-3.28) | 1.91 (1.24-2.96) |
|  | *BRAF+*dMMR | 10 |  | 9 (90.0) | 1.16 (0.58-2.32) | 0.91 (0.44-1.88) |  | 10 (100.0) | 1.20 (0.62-2.32) | 0.97 (0.48-1.93) |
|  | Other+pMMR | 35 |  | 31 (88.6) | 1.23 (0.82-1.85) | 1.50 (0.99-2.27) |  | 35 (100.0) | 1.25 (0.85-1.84) | 1.53 (1.03-2.26) |
|  | Other+dMMR | 6 |  | 3 (50.0) | 0.23 (0.07-0.74) | 0.13 (0.04-0.42) |  | 6 (100.0) | 0.40 (0.17-0.93) | 0.23 (0.09-0.54) |

**Supplementary Table S7 –** Frequencies of the mutational subgroups, stratified on pTNM stage and Warburg-subtype (Warburg-low, -moderate, -high).

|  | | **CRC** | | | | |  |
| --- | --- | --- | --- | --- | --- | --- | --- |
|  | | **Total** | **Warburg-low** | **Warburg-moderate** | | **Warburg-high** | |
| **Colorectal** | |  |  |  |  | |  |
|  | All-wild-type+pMMR | 827 (36.5) | 285 (44.1) | 300 (36.6) | 242 (30.2) | |  |
|  | *KRAS_mut_+*pMMR | 554 (24.4) | 128 (19.8) | 226 (27.6) | 200 (24.9) | |  |
|  | *KRAS_mut_+PIK3CA_mut_+*pMMR | 168 (7.4) | 48 (7.4) | 69 (8.4) | 51 (6.4) | |  |
|  | *PIK3CA_mut_+*pMMR | 118 (5.2) | 43 (6.7) | 36 (4.4) | 39 (4.9) | |  |
|  | *BRAF_mut_+*pMMR | 144 (6.4) | 24 (3.7) | 38 (4.6) | 82 (10.2) | |  |
|  | *BRAF_mut_+*dMMR | 132 (5.8) | 32 (5.0) | 39 (4.8) | 61 (7.6) | |  |
|  | Other+pMMR | 211 (9.3) | 63 (9.8) | 75 (9.2) | 73 (9.1) | |  |
|  | Other+dMMR | 114 (5.0) | 23 (3.6) | 37 (4.5) | 54 (6.7) | |  |
|  |  |  |  |  |  | |  |
| **pTNM stage I** | |  |  |  |  | |  |
|  | All-wild-type+pMMR | 192 (43.2) | 75 (46.9) | 66 (39.8) | 51 (43.2) | |  |
|  | *KRAS_mut_+*pMMR | 111 (25.0) | 38 (23.8) | 46 (27.7) | 27 (22.9) | |  |
|  | *KRAS_mut_+PIK3CA_mut_+*pMMR | 26 (5.9) | 9 (5.6) | 11 (6.6) | 6 (5.1) | |  |
|  | *PIK3CA_mut_+*pMMR | 17 (3.8) | 8 (5.0) | 3 (1.8) | 6 (5.1) | |  |
|  | *BRAF_mut_+*pMMR | 10 (2.3) | 3 (1.9) | 2 (1.2) | 5 (4.2) | |  |
|  | *BRAF_mut_+*dMMR | 19 (4.3) | 4 (2.5) | 9 (5.4) | 6 (5.1) | |  |
|  | Other+pMMR | 51 (11.5) | 15 (9.4) | 22 (13.3) | 14 (11.9) | |  |
|  | Other+dMMR | 18 (4.1) | 8 (5.0) | 7 (4.2) | 3 (2.5) | |  |
| **pTNM stage II** | |  |  |  |  | |  |
|  | All-wild-type+pMMR | 298 (34.9) | 99 (42.5) | 109 (37.1) | 90 (27.6) | |  |
|  | *KRAS_mut_+*pMMR | 179 (21.0) | 40 (17.2) | 72 (24.5) | 67 (20.6) | |  |
|  | *KRAS_mut_+PIK3CA_mut_+*pMMR | 73 (8.6) | 18 (7.7) | 32 (10.9) | 23 (7.1) | |  |
|  | *PIK3CA_mut_+*pMMR | 50 (5.9) | 18 (7.7) | 12 (4.1) | 20 (6.1) | |  |
|  | *BRAF_mut_+*pMMR | 45 (5.3) | 7 (3.0) | 9 (3.1) | 29 (8.9) | |  |
|  | *BRAF_mut_+*dMMR | 65 (7.6) | 16 (6.9) | 16 (5.4) | 33 (10.1) | |  |
|  | Other+pMMR | 72 (8.4) | 23 (9.9) | 24 (8.2) | 25 (7.7) | |  |
|  | Other+dMMR | 71 (8.3) | 12 (5.2) | 20 (6.8) | 39 (12.0) | |  |
| **pTNM stage III** | |  |  |  |  | |  |
|  | All-wild-type+pMMR | 214 (36.0) | 71 (45.5) | 88 (39.5) | 55 (25.5) | |  |
|  | *KRAS_mut_+*pMMR | 150 (25.2) | 29 (18.6) | 63 (28.3) | 58 (26.9) | |  |
|  | *KRAS_mut_+PIK3CA_mut_+*pMMR | 39 (6.6) | 13 (8.3) | 11 (4.9) | 15 (6.9) | |  |
|  | *PIK3CA_mut_+*pMMR | 30 (5.0) | 11 (7.1) | 10 (4.5) | 9 (4.2) | |  |
|  | *BRAF_mut_+*pMMR | 58 (9.8) | 8 (5.1) | 16 (7.2) | 34 (15.7) | |  |
|  | *BRAF_mut_+*dMMR | 35 (5.9) | 9 (5.8) | 11 (4.9) | 15 (6.9) | |  |
|  | Other+pMMR | 50 (8.4) | 13 (8.3) | 17 (7.6) | 20 (9.3) | |  |
|  | Other+dMMR | 19 (3.2) | 2 (1.3) | 7 (3.1) | 10 (4.6) | |  |
| **pTNM stage IV** | |  |  |  |  | |  |
|  | All-wild-type+pMMR | 96 (30.0) | 27 (34.2) | 28 (24.4) | 41 (32.5) | |  |
|  | *KRAS_mut_+*pMMR | 104 (32.5) | 19 (24.1) | 40 (34.8) | 45 (35.7) | |  |
|  | *KRAS_mut_+PIK3CA_mut_+*pMMR | 27 (8.4) | 7 (8.9) | 14 (12.2) | 6 (4.8) | |  |
|  | *PIK3CA_mut_+*pMMR | 17 (5.3) | 5 (6.3) | 9 (7.8) | 3 (2.4) | |  |
|  | *BRAF_mut_+*pMMR | 27 (8.4) | 6 (7.6) | 8 (7.0) | 13 (10.3) | |  |
|  | *BRAF_mut_+*dMMR | 10 (3.1) | 3 (3.8) | 2 (1.7) | 5 (4.0) | |  |
|  | Other+pMMR | 33 (10.3) | 11 (13.9) | 11 (9.6) | 11 (8.7) | |  |
|  | Other+dMMR | 6 (1.9) | 1 (1.3) | 3 (2.6) | 2 (1.6) | |  |


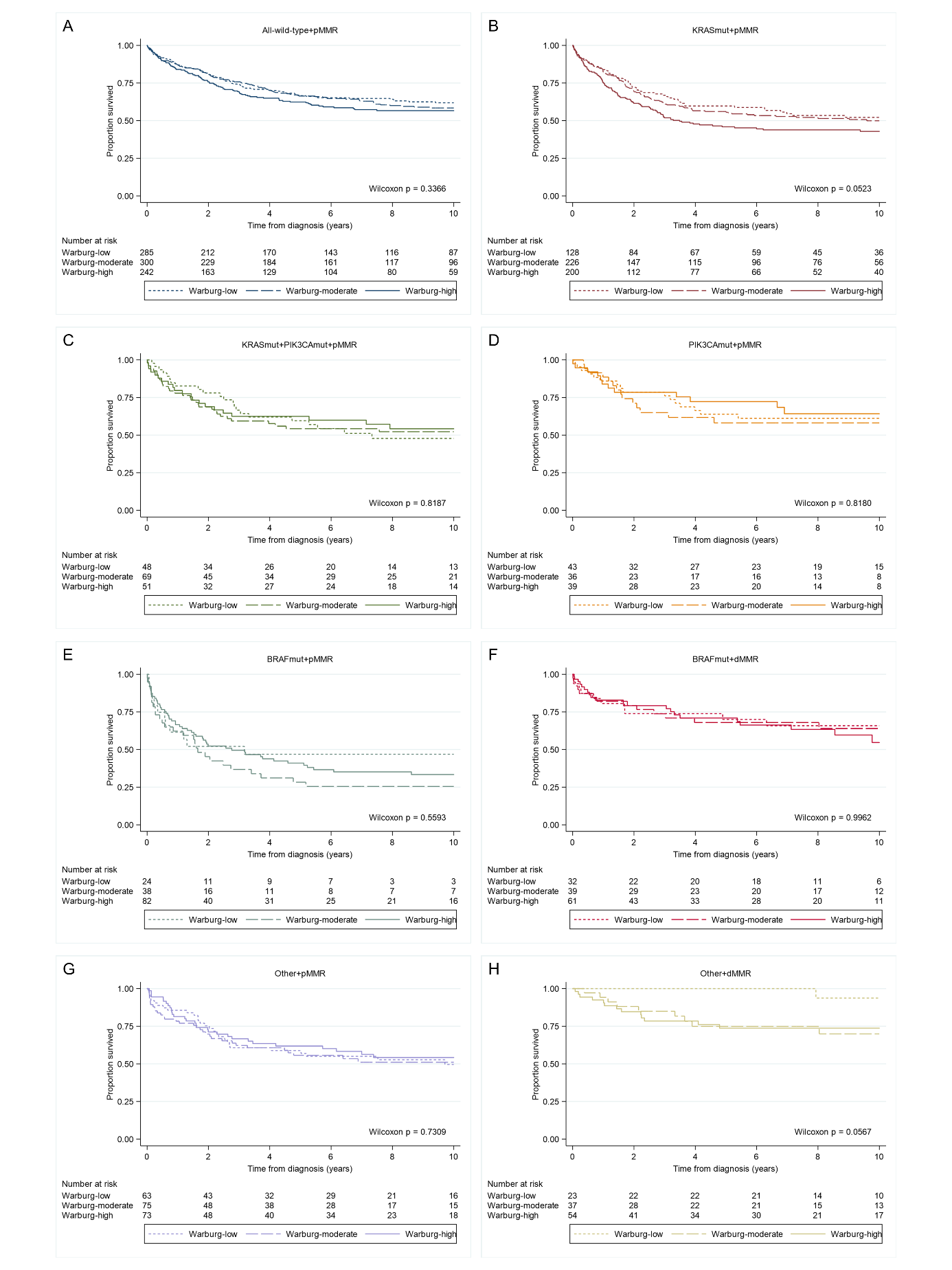


**Supplementary Figure S1** – Kaplan-Meier curves showing CRC-specific survival of Warburg-subtypes, according to mutational subgroup: (A) all-wild-type+pMMR, (B) KRAS_mut_+pMMR, (C) KRAS_mut_+PIK3CA_mut_+pMMR, (D) PIK3CA_mut_+pMMR, (E) BRAF_mut_+pMMR, (F) BRAF_mut_+dMMR, (G) other+pMMR, (H) other+dMMR.
